# Early Eocene Palynofloral Diversity and *Nothofagus* Niche Modeling Across Western Gondwana

**DOI:** 10.3390/plants15071122

**Published:** 2026-04-07

**Authors:** Luis Felipe Hinojosa, Francy Milena Carvajal, Mirta Quattrocchio, Damián A. Fernández, María Fernanda Pérez

**Affiliations:** 1 Laboratorio de Paleoecología, Facultad de Ciencias, Universidad de Chile, Santiago 7800003, Chile; 2 Center for Tropical Paleoecology and Archaeology, Smithsonian Tropical Research Institute, Panama City 0843-03092, Panama; frmcarvajal@gmail.com; 3 Departamento de Geología, Universidad Nacional del Sur, Bahía Blanca B8000, Argentina; mquattro@criba.edu.ar; 4 Centro Austral de Investigaciones Científicas (CADIC-CONICET), Ushuaia V9410, Argentina; 5 Instituto de Ciencias Polares, Ambiente y Recursos Naturales, Universidad Nacional de Tierra del Fuego (ICPA-UNTDF), Ushuaia V9410, Argentina; dafernandez@untdf.edu.ar; 6 Facultad de Ciencias Biológicas, Pontificia Universidad Católica de Chile, Santiago 8331150, Chile; mperezt@bio.puc.cl

**Keywords:** early Eocene, Nothofagaceae, diversity, niche model

## Abstract

During warm intervals such as the Early Eocene, megathermal vegetation belts expanded toward higher latitudes, displacing mesothermal and microthermal biota. Here, we examine the diversity and paleoclimate of the Early Eocene Ligorio Márquez Formation (LMF) in the context of other Paleogene Patagonian palynofloras, and we model the potential distribution of *Nothofagus* using Early Eocene climate simulations. From 35 processed samples, 20 yielded palynomorphs and 85 morphospecies were distinguished. We hypothesize that species richness in the LMF is comparable to other Eocene microfloras, and that climate models will confirm mesothermal conditions for this formation while identifying western Gondwana as the primary region of climatic suitability for *Nothofagus*. Our results indicate that the LMF hosted a diverse flora under mesothermal, humid-temperate conditions (Köppen–Geiger climate Cfa, within the broader Cf no-dry-season regime). Ecological niche modeling further indicates that western Gondwana (South America, the Antarctic Peninsula, New Zealand, and Australia) provided broadly suitable climatic conditions for *Nothofagus*. In Experiment 1 (modern-to-Eocene transfer), Maxnet models showed high discriminatory power (AUC_test = 0.86–0.88) with low omission at P10 (OR_P10 = 0.099–0.128). In Experiment 2 (Eocene-to-Eocene calibration), performance was consistently high across GCMs (AUC_test = 0.87–0.98; OR_P10 = 0.091–0.182). However, conditions across Antarctica were likely challenging, limiting its effectiveness as a dispersal corridor during the Eocene. Finally, our results suggest that the ancient South Pacific High influenced the northern distributional limit of *Nothofagus* in South America.

## 1. Introduction

Throughout the Cenozoic, the climate transitioned from a “hothouse” to an “icehouse”, a period marked by ice sheets at both poles, driven by geological and orbital processes [1,2]. The marine δ^18^O record exhibits several steps and peaks that mirror global climatic changes, encompassing ice-sheet expansion and retreat and a significant decrease in δ^13^C within continental sediments [2]. The Hothouse occurred between the Paleocene-Eocene Thermal Maximum at 56 Ma and the end of the Early Eocene Climate Optimum (EECO) at 47 Ma, when temperatures were more than 10 °C warmer than they are today and displayed greater amplitude variability [1]. During this period, the pCO_2_ reached values up to 720 ppm with a peak of 1600 ppm at ~51 Ma during the EECO [3].

It has been postulated that during warmer periods, such as the Early Eocene, vegetational belts with biota adapted to megathermal climates (i.e., Mean Annual Temperature (MAT) > 22 °C, ref. [4] would expand their distribution toward higher latitudes. This expansion would result in the displacement of mesothermal (MAT 14–22 °C)—microthermal (MAT < 14 °C) biota to even higher latitudes [5,6,7]. Under this scenario, species richness, which refers to the diversity of species within a particular area, would be greater during warmer periods in mid-latitudes [5,7,8,9].

Fossil records from the South American tropics show increased plant diversity and origination rates between the Upper Paleocene and Lower Eocene flora [10,11]. This trend displays a diversity peak during the Paleogene, with values surpassing those of Holocene records in the same area [10,12]. Simultaneously, in southern South America, fossil evidence reveals high levels of diversity in paleontological records during the Eocene. The number of mammalian genera increased significantly from preceding levels at the onset of the Eocene Climatic Optimum [13]. Plant–insect associations in the Patagonian Eocene ecosystem demonstrate heightened richness, comparable to modern associations in tropical and subtropical forests [14]. Furthermore, fossil flora from Argentina’s Patagonia, specifically at Laguna del Hunco and Río Pichileufú, provide compelling macrofossil evidence for the ancient history of high plant diversity at mid-high latitudes during the Eocene of South America [14,15,16].

Numerous models have been proposed to elucidate the relationship between vegetation response and climatic changes in southern South America throughout the Paleogene–early Neogene [17,18,19,20,21,22,23]. Paleophytogeographical models indicate a temporal and spatial succession of distinct paleofloristic assemblages, ranging from Gondwanan to Mixed and Subtropical Neogene floras [18,21,24].

The succession of these floras is closely tied to at least three climatic scenarios. First, mesothermal conditions prevailed during the Paleocene–early Eocene climatic optimum. Second, the Eocene–Oligocene transition saw temperate and drier conditions due to Antarctic glaciation. Third, a cooling trend marked the end of the Neogene epoch, punctuated by a warm climatic optimum in the Middle Miocene, characterized by a temperature increase of 6–9 °C compared to the Oligocene epoch [18,21].

The paleofloristic sequence described above has been replicated in the palynological record during the Cenozoic in Patagonia [21]. From the Paleocene to Early Eocene, a Gondwana Paleoflora inhabited southern South America. This flora included lineages such as Podocarpaceae, Araucariaceae, Cunoniaceae, and Proteaceae, along with tropical-subtropical taxa like Arecaceae and Myricaceae.

A significant shift occurred in the Middle Eocene, marked by a rapid transition from the Gondwana Paleoflora to a Mixed Paleoflora, which persisted until the Oligocene. This transition featured the prevalence of the Nothofagaceae family in the palynological record and the gradual replacement of mega-mesothermal communities by microthermal rainforests [25].

By the Late Oligocene to Early Miocene, warm climates facilitated the southward dispersal of some neotropical elements. These included palms, *Cupania*, *Alchornea*, Rubiaceae, and Combretaceae, introducing megathermal elements to the existing local floras. The eventual disappearance of mega-mesothermal elements in Patagonia characterized Miocene palynofloras. This period also saw increased diversity and abundance of xerophytic taxa, such as Asteraceae, Chenopodiaceae, and Convolvulaceae [21,25].

The Ligorio Márquez Formation (LMF), named after the coal mine located in Chilean Patagonia (46°45′ S; 71°50′ W), records the earliest deposits of the lower Paleogene identified in the Chilean Patagonia region [26,27] (see Section 4 for age discussion).

Ligorio Marquez flora thrived in warm, humid, frost-free, mesothermal climates during the Early Eocene. At that time, the Mean Annual Temperature was approximately 17° to 20 °C, with Annual Precipitation exceeding 1500 mm. The presence of *Nothofagus* in the LMF marks the northern distribution limit of the taxon in South America during the Early Eocene. This suggests that floras with the iconic Gondwana taxa *Nothofagus* occupied the southern landmasses of western Gondwana during the Hothouse time of the Early Eocene, including areas such as southern South America, Antarctica, and Australia. In contrast, species from floras without *Nothofagus* (such as the one from Laguna del Hunco) not only expanded their distribution toward higher latitudes but also reached their southernmost limits during this period [23,28].

The development of standardized paleoclimate modeling frameworks for key moments of Earth’s history, such as the Early Eocene, which generate gridded climate fields under harmonized boundary conditions and multiple CO_2_ forcings [29]. Together with emerging AI-enabled analytical workflows that facilitate synthesis across heterogeneous biodiversity data [30], these simulations provide a valuable, independent line of evidence to evaluate paleoclimate reconstructions for southern South America and to conduct spatially explicit tests of paleobiogeographic hypotheses that help us understand the effects of latitudinal shifts in thermal belts and changes in habitat suitability for key lineages, such as Gondwana taxa like *Nothofagus.*

Building on this framework, the Ligorio Márquez Formation (LMF) provides a well-constrained case study to evaluate whether independent paleoclimate simulations and biotic data converge on a consistent ecological and biogeographic interpretation. Here we ask whether the Early Eocene climate, under both 3× and 6× pre-industrial pCO_2_ scenarios, provides a coherent mechanistic framework to explain (i) the ecological signal recorded by the LMF palynoflora and (ii) the spatial distribution of climatically suitable habitats for *Nothofagus* across Gondwana.

To address this question, we (1) compare palynological diversity of the LMF with other Eocene Patagonian assemblages using rarefaction-based inference; (2) evaluate published LMF paleoclimate estimates against DeepMIP–Eocene simulations [29] under 3× pre-industrial CO_2_ (~800 ppm; seven models) and 6× pre-industrial (~1600 ppm; three models, approximating EECO conditions); and (3) develop ecological niche models for *Nothofagus* and project habitat suitability onto Early Eocene climates across Gondwana.

We hypothesized that the Early Eocene climate generated mesothermal, humid conditions at LMF and a geographically structured belt of suitable habitats for *Nothofagus* across western Gondwana. This hypothesis yields three linked predictions: (1) LMF species richness is comparable to other highly diverse Eocene Patagonian palynofloras (i.e., no statistical difference in rarefied richness among “Hothouse” assemblages); (2) DeepMIP simulations under both 3× and 6× pre-industrial pCO_2_ reproduce mesothermal conditions at LMF (mean annual temperature between 14 °C and 22 °C, with annual precipitation exceeding 1000 mm); and (3) projected climatic suitability for *Nothofagus* is concentrated in western Gondwana, specifically southern South America, Antarctica, Australia, and New Zealand, thereby constraining the most plausible regions for dispersal and persistence during the Early Eocene.

## 2. Results

### 2.1. Rarefied Richness Across Eocene Hothouse Assemblages

**Figure 1:**
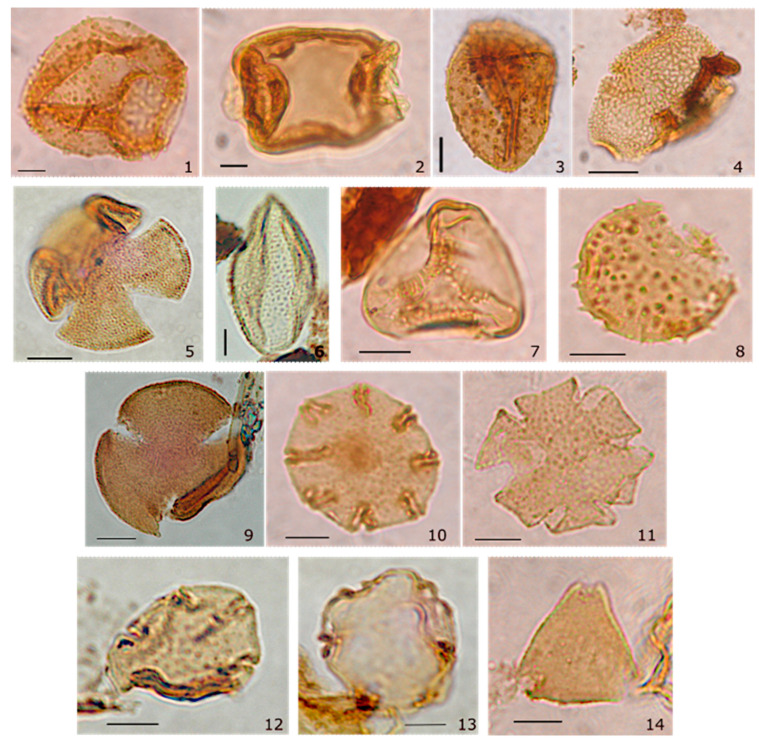
Microphotographs of selected palynomorphs with their possible modern analogs from the Early Eocene of Ligorio Marquez Formation. Sample number. Scale bar = 10 μm. (1) *Dilwynites granulatus* Harris 1965, Araucariaceae. (2) *Phyllocladidites mawsonii* (Cookson 1947) ex Couper 1953, Podocarpaceae. (3) *Mauritiidites francisco var. minutus* Van der Hammen & Garcia 1966, Arecaceae (4) *Bombacacidites* sp., Bombacaceae. (5) *Retistephanocolpites regularis* Hoeken-Klinkenberg 1966, Bombacaceae. (6) *Liliacidites variegatus* Couper 1953, Liliaceae. (7) *Gothanipollis perplexus* Pocknal & Mildenhall 1984, Loranthaceae. (8) *Malvacipollis diversus* Harris 1965, Malvaceae. (9) *Tricolpites* cf. *reticulata* Cookson 1947, Gunneraceae. (10) *Nothofagidites kaitangataensis* (Te punga) Romero 1973, Nothofagaceae. (11) *N. dorotensis* Romero 1973, Nothofagaceae. (12) *N. acromegacanthus* Menéndez & Caccavari 1975, Nothofagaceae. (13) *Nothofagus* fusca group, Nothofagaceae. (14) *Proteacidites* cf. *subscabratus* Couper 1960, Proteaceae.

From the 35 palynological samples processed from the LMF, 20 yielded palynomorphs. A total of 85 morphospecies were distinguished in LMF, grouped as follows: 30 morphospecies of spores, 13 species of gymnosperm pollen grains, and 42 angiosperms. Other indeterminate spores and grain corresponding to algae spores, fungi, gymnosperm, and angiosperm were recovered from LMF (Figure 1, Support Information Figure S1). The total of morphospecies for LMF, considering the presence reported previously [31,32], reaches 119 taxa (Tables S1 and S2 in Support Information).

**Figure 2:**
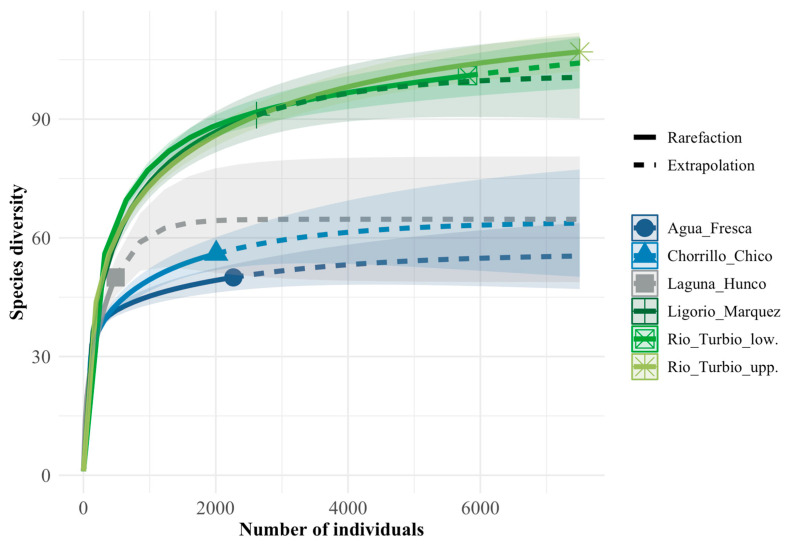
Abundance-based rarefaction (solid lines) and extrapolation (dashed lines) curves comparing palynological morphospecies richness among the Ligorio Marquez Formation and other Paleogene Patagonian assemblages (Rio Turbio, Laguna del Hunco, Agua Fresca, and Chorrillo Chico). Curves were computed from abundance (count) data. Shaded bands indicate 95% confidence intervals (bootstrap).

The rarefaction analysis (Figure 2) shows that LMF has no difference in species richness compared to the Rio Turbio Formation. In contrast, LMF shows higher richness than the Paleocene–early Eocene assemblages from Chorrillo Chico and Agua Fresca. Laguna del Hunco appears to have lower richness, but it could not be included in the standardized pairwise tests (see below).

Pairwise comparisons of rarefied species richness (q = 0) standardized to m_0_ = 2007 individuals are summarized in Table 1. Using Δ = S_A(m_0_) − S_B(m_0_), Ligorio Márquez (LMF) and Río Turbio lower (RT_low) show higher rarefied richness than Chorrillo Chico (CC) and Agua Fresca (AF) (Δ = 28.6–38.7; all *p* < 0.005 and *p*_Bonf < 0.005). Río Turbio upper (RT_upp) also shows higher rarefied richness than CC and AF (CC vs. RTsup: Δ = −32.4; AF vs. RTsup: Δ = −37.4; both *p* < 0.005 and *p*_Bonf < 0.005). The remaining contrasts were not significant after Bonferroni correction (Table 1). Assemblage-level sampling effort and richness (N, S_obs, and S(m0) with 95% bootstrap CIs) are reported in Table S3.

Finally, Laguna del Hunco was excluded from the standardized pairwise rarefaction comparisons because its total abundance fell below the predefined minimum-sample-size threshold; therefore, it could not be compared at m_0_ = 2007 without extrapolation.

### 2.2. Mesothermal, Humid Conditions at LMF in 3× and 6× pCO_2_ DeepMIP Simulations

**Figure 3:**
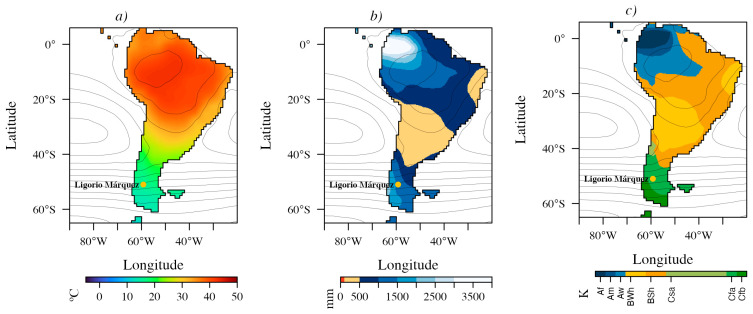
Ensemble-mean early Eocene climate over South America from DeepMIP simulations under 3 × CO_2_. (**a**) Mean annual temperature, (**b**) annual precipitation, and (**c**) Köppen–Geiger climate classification. Contours show mean sea-level pressure (hPa).

**Figure 4:**
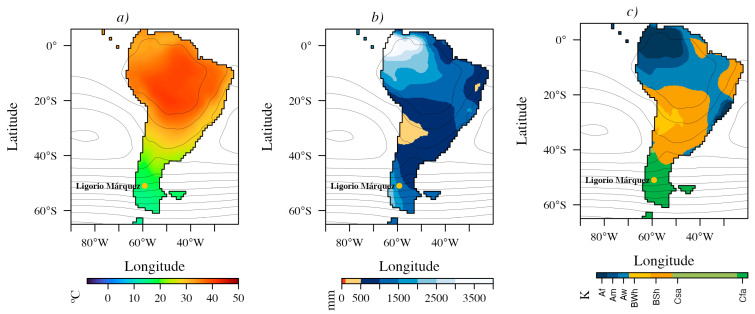
Ensemble-mean early Eocene climate over South America from DeepMIP simulations under 6 × CO_2_. (**a**) Mean annual temperature, (**b**) annual precipitation, and (**c**) Köppen–Geiger climate classification. Contours show mean sea-level pressure (hPa).

The ensemble means of seven 3 × CO_2_ and three 6 × CO_2_ DeepMIP simulations (Figure 3 and Figure 4) indicate predominantly megathermal conditions (mean annual temperature, MAT, 22–40 °C) across the low to mid-latitudes of South America (6° N to ~40° S), grading into mesothermal climates (14–22 °C) south of 40° S. In the 3 × CO_2_ simulations, microthermal conditions (MAT < 14 °C) emerge at the southern tip of South America (~61° S) and prevail across most of Antarctica. In the 6 × CO_2_ simulations, this transition shifts farther south, into the Antarctic Peninsula (~64° S).

Both ensembles also resolve an arid belt near ~30° S, consistent with the influence of the Southeast Pacific Subtropical Anticyclone. Relative to the 3 × CO_2_ ensemble, the 6 × CO_2_ ensemble yields generally warmer conditions over the continent and increased moisture availability around ~30° S, with annual precipitation of ~500–1000 mm (Figure 4b). In contrast, south of ~40° S annual precipitation is consistently high, ranging from ~1000 to 2500 mm under 3 × CO_2_ and from ~1000 to 3000 mm under 6 × CO_2_ (Figure 3b and Figure 4b).

Köppen–Geiger classifications for the Early Eocene of South America are dominated by three broad climate groups: Tropical (A), Arid (B), and Temperate (C), whose spatial extents vary between pCO_2_ scenarios (Figure 3c and Figure 4c). Under 3 × CO_2_ conditions (Figure 3c), tropical climates (A) were confined to the northeast, ranging from tropical rainforest without a dry season (Af) to tropical savanna with a winter dry season (Aw) toward the southeast. Arid climates (B) covered much of the continent, with a hyper-arid core (BWh) centered near ~30° S and bordered by a semi-arid belt (BSh) extending to ~36° S along the western margin and ~39° S along the eastern margin. South of these latitudes, temperate climates (C) prevailed across southern South America. Humid subtropical conditions (Cfa) extended to ~50–55° S, transitioning poleward into oceanic/subtropical highland climates (Cfb), which reached the Antarctic Peninsula (Figure 3c). Along the western margin, between ~36° S and 42° S, climates were classified as hot-summer Mediterranean (Csa). Overall, Temperate mesothermal (C) climates were widespread within the westerlies-dominated belt between the subtropical high-pressure and subpolar low-pressure systems (Figure 3 and Figure 4).

Under 6 × CO_2_ conditions (Figure 4c), tropical (A) climates expanded into temperate latitudes along the eastern margin, the BWh hyper-arid zone near ~30° S contracted, and humid subtropical (Cfa) climates became more extensive, reaching as far south as the Antarctic Peninsula. The Ligorio Márquez locality falls within the Cfa climate type in both ensemble reconstructions.

**Figure 5:**
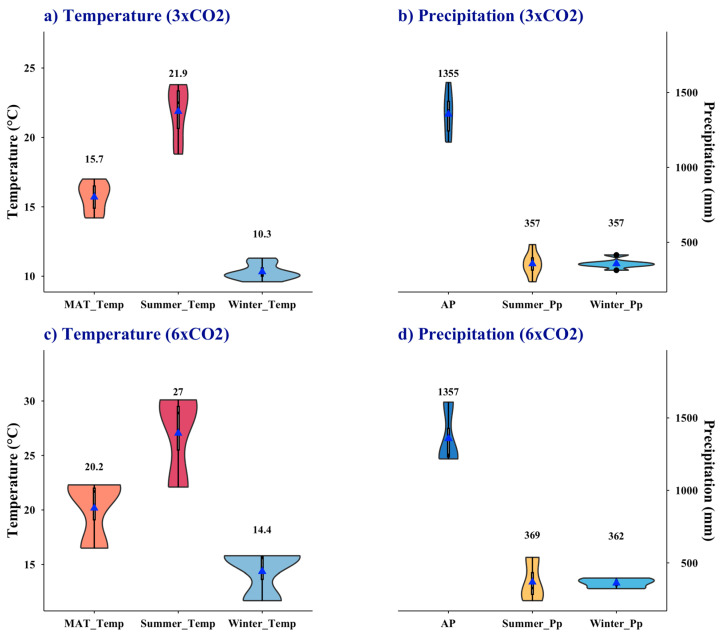
Paleoclimate at the Ligorio Márquez Formation inferred from DeepMIP simulations. (**a**,**b**) Conditions at the LMF under 3 × CO_2_; (**c**,**d**) conditions under 6 × CO_2_. Temperature variables: MAT, mean annual temperature; Summer_Temp, DJF (December–February) mean temperature in the Southern Hemisphere; Winter_Temp, JJA (June–August) mean temperature in the Southern Hemisphere. Precipitation variables: AP, annual precipitation; Summer_Pp, DJF precipitation; Winter_Pp, JJA precipitation.

The values extracted under the 3 × CO_2_ and 6 × CO_2_ scenarios confirm the mesothermal character of the LMF (Figure 5). Under the 3 × CO_2_ models, the mean annual temperature (MAT) was 15.7 °C, with winter and summer averages of 10.3 °C and 21.9 °C, respectively (Figure 5a). Annual precipitation reached 1355 mm, with similar amounts in winter and summer (357 mm) (Figure 5b). Under the 6 × CO_2_ models, MAT increased to 20.2 °C, with winter and summer averages of 14.4 °C and 27 °C, respectively (Figure 5c). Annual precipitation was 1357 mm, again showing comparable values in winter (362 mm) and summer (369 mm) (Figure 5d).

### 2.3. Nothofagus Ecological Niche Models and Early Eocene Suitability Across Gondwana

**Figure 6:**
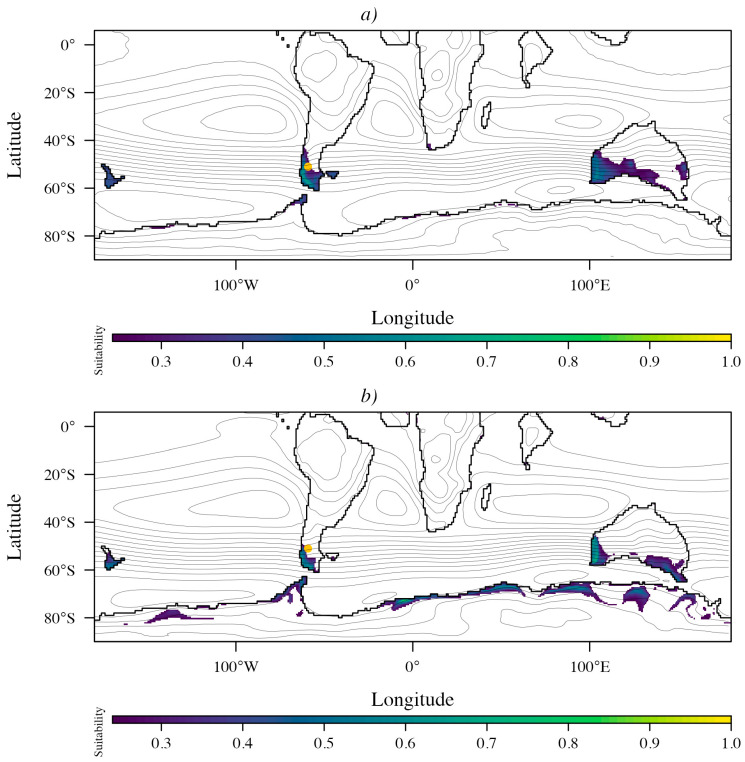
Ensemble-mean Nothofagus suitability across Gondwana during the Eocene projected from modern occurrence records. Colors represent continuous predicted suitability (cloglog), with the color scale anchored near the 10th percentile training presence threshold (P10 = 0.25). (**a**) Suitability based on the mean of seven 3 × CO_2_ GCM simulations. (**b**) Suitability based on the mean of three 6 × CO_2_ GCM simulations. Contour lines indicate Eocene annual mean sea-level pressure isobars. The yellow dot corresponds to the Ligorio Marquez Formation.

Figure 6 illustrates the ensemble-mean suitability of *Nothofagus* during the Eocene, projected from its current distribution under the 3 × CO_2_ (Figure 6a) and 6 × CO_2_ (Figure 6b) scenarios. Model performance was consistently high across DeepMIP general circulation model (GCM) specific calibrations (AUC = 0.86–0.88 and omission rate = 0.099–0.128; Table 2), with a regularization multiplier of 1 in all cases. Selected feature sets were dominated by linear hinge (lh), with more complex configurations (linear + quadratic + hinge (lqh) and linear + quadratic + product + hinge (lqph)) retained in a subset of models; CESM, GFDL, and INMCM were used for the 6 × CO_2_ Eocene projections. The model signal was most consistently associated with hydroclimatic seasonality and thermal extremes (Table 3). JJA precipitation was the leading predictor in CESM (both 3 × CO_2_ and 6 × CO_2_) and in HADCM3BL (3 × CO_2_) and ranked second in HADCM3B and IPSL. Temperature-related predictors were likewise recurrent, including DJF mean temperature (rank 2 in CESM; rank 1 in MIROC; and rank 1 in INMCM under 6 × CO_2_), mean annual temperature (rank 1 in HADCM3B), and temperature of the warmest month (rank 1 in COSMOS, GFDL, and GFDL under 6 × CO_2_). Several models also emphasized variables reflecting evaporative demand or moisture balance, such as DJF evaporation (HADCM3B), JJA EP (COSMOS, GFDL, MIROC), and EP min (IPSL), whereas orography emerged as an important constraint in some cases (notably GFDL and MIROC).

Under the 3 × CO_2_ scenario, suitable conditions for *Nothofagus* (probability P10 > 0.24) were restricted to southwestern South America (south of ~40° S); western Australia, New Zealand, and the Antarctic Peninsula (Figure 6a). Under the 6 × CO_2_ scenario, suitable areas expanded toward Antarctica but did not form a continuous corridor between South America and Australia, and only scattered, isolated patches of suitability were projected across the continent (Figure 6b). In both scenarios, the highest suitability was largely concentrated within regions influenced by the Southern Hemisphere westerlies (Figure 6).

**Figure 7:**
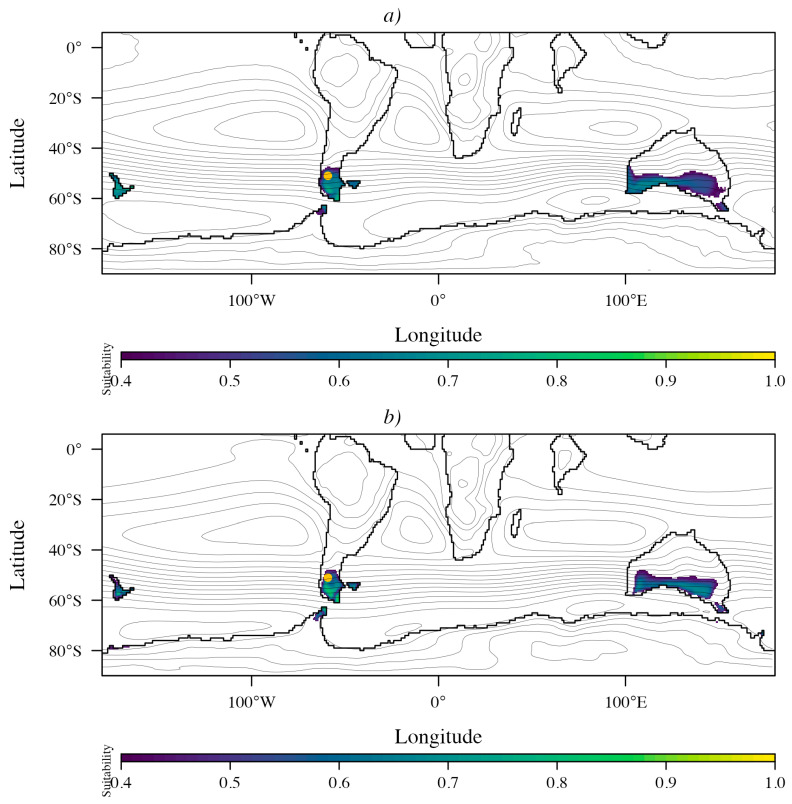
Ensemble-mean *Nothofagus* suitability across Gondwana during the Eocene projected from Eocene occurrence records. Colors represent continuous predicted suitability (cloglog), with the color scale anchored near the 10th percentile training presence threshold (P10 = 0.5). (**a**) Suitability based on the mean of seven 3 × CO_2_ GCM simulations. (**b**) Suitability based on the mean of three 6 × CO_2_ GCM simulations. Contour lines indicate Eocene annual mean sea-level pressure isobars. The yellow dot corresponds to the Ligorio Marquez Formation.

Figure 7 illustrates the ensemble-mean suitability of *Nothofagus* during the Eocene, projected from its Eocene occurrence records under the 3 × CO_2_ (Figure 7a) and 6 × CO_2_ (Figure 7b) scenarios. Model performance was consistently high across DeepMIP general circulation model (GCM)–specific calibrations (AUC = 0.89–0.98 and omission rate = 0.091–0.182; Table 4), with modest variation in model complexity reflected by the selected feature classes (l, lh, lq, lqph) and regularization multipliers (1–3; Table 4). Variable-importance rankings (Table 5) showed that the dominant predictors shifted toward stronger topographic and seasonal-temperature control across multiple GCMs. DJF mean temperature clearly dominated CESM and COSMOS (both scenarios), while orography ranked first in HADCM3B, HADCM3BL, and MIROC (3 × CO_2_) and ranked second in several other GCMs, indicating an increased sensitivity to physiographic structure in the fossil-calibrated reconstructions. GFDL remained primarily driven by the temperature of the warmest month (rank 1 in both scenarios), with orography and EP max consistently ranking second and third, respectively. In IPSL, JJA precipitation was overwhelmingly dominant (rank 1), whereas INMCM under 6 × CO_2_ showed a distinct response in which temperature range (Tmax–Tmin) and JJA mean temperature were the leading predictors, again coupled with EP max.

Under the 3 × CO_2_ scenario, suitability (probability P10 > 0.5) was concentrated within the westerlies-influenced belt south of ~40° S, including southern South America, southern Australia, New Zealand, and the Antarctic Peninsula (Figure 7a). Under the 6 × CO_2_ scenario, suitable areas expanded within the Antarctic Peninsula and included an isolated region in eastern Antarctica; however, Antarctica did not form a continuous corridor between South America and Australia, and only scattered, isolated patches of suitability were projected across the continent (Figure 7b).

**Figure 8:**
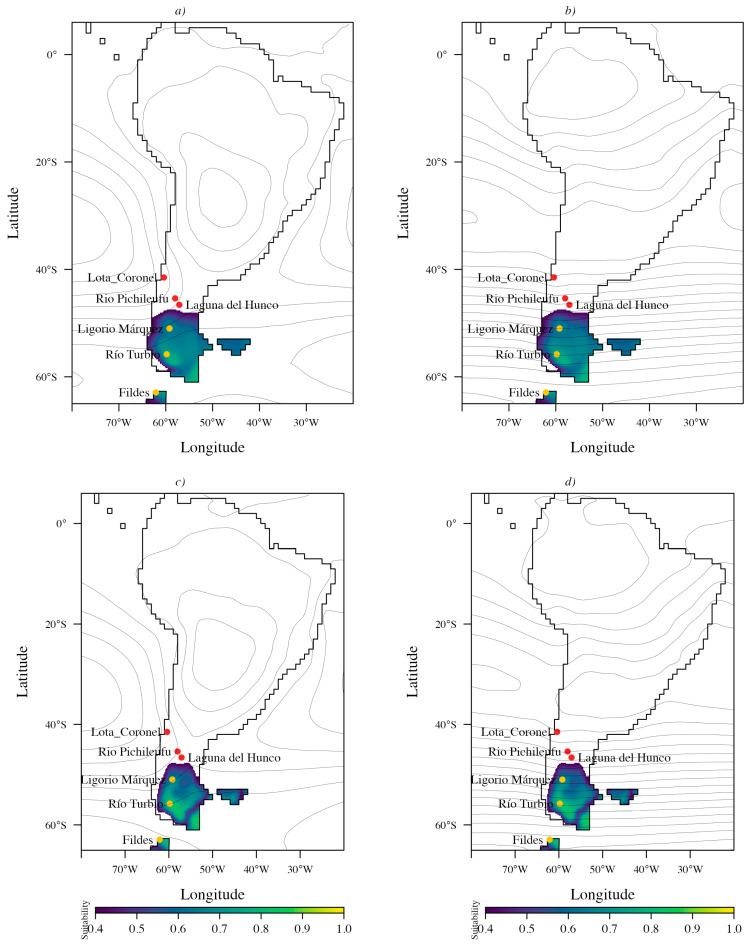
*Nothofagus* suitability (10th percentile training presence threshold (P10 = 0.5)) during the Early Eocene in South America and the Antarctic Peninsula. (**a**) Model under 3 × CO_2_, associated with winter pressure circulation; (**b**) Model under 3 × CO_2_, associated with summer pressure circulation; (**c**) Model under 6 × CO_2_, associated with winter pressure circulation; (**d**) Model under 6 × CO_2_, associated with summer pressure circulation. Yellow dots represent fossil floras with *Nothofagus*, and red dots represent floras without *Nothofagus*. Lines indicate isobaric pressure during JJA (summer; panels (**a**,**c**)) and DJF (winter; panels (**b**,**d**)).

Figure 8 illustrates the probability of *Nothofagus* occurrence in South America and the Antarctic Peninsula in relation to the pressure system during winter (Figure 8a,c) and summer (Figure 8b,d). Panels a and b correspond to the mean of the 3 × CO_2_ models, while panels c and d correspond to the mean of the 6 × CO_2_ models. *Nothofagus* Eocene distribution is associated mainly with the westerlies belt, with a northern limit restricted by the presence of the South American anticyclone during the summertime. This transition area will lie between South America’s subtropical and temperate climates and will separate the Eocene floras with and without *Nothofagus*.

## 3. Discussion

The mid-latitudes south of ~40° S during the Eocene was predominantly mesothermal (Figure 3 and Figure 4), supporting highly diverse floras. Laguna del Hunco, among the most diverse Eocene macrofloras, is linked to EECO [16]. Paleoclimate estimates for this flora indicate mesothermal conditions, with a mean annual temperature of 16.6 °C and annual precipitation of 1140 mm. Similarly, high diversity is documented in the Rio Turbio microflora [8,16,33], with mean annual temperatures of ~16 °C and annual precipitation of 1540 mm [18,21]. For Ligorio Márquez flora, DeepMIP-based values are ~3.4 °C cooler under 3 × CO_2_ (MAT = 15.7 °C; range: 14.2–17.0 °C; Figure 5a) than previous estimates (19.1 °C; range: 17.2–20.9 °C) reported by Hinojosa et al. [28] and Quattrocchio et al. [21]. By contrast, the earlier estimates are more consistent with the 6 × CO_2_ scenario (20.2 °C; range: 16.5–22.3 °C), although all values remain within the mesothermal range expected for the Early Eocene in this region.

Our diversity findings from the LMF (Figure 1 and Figure 2 and Figure S1) are consistent with the influence of the Paleocene/Eocene–Early Eocene Hothouse climate on southern South American biodiversity, with species richness comparable to that of the most diverse Patagonian microfloras reported to date [33]. In contrast, the LMF appears more diverse than the Paleocene–Early Eocene microfloras of Chorrillo Chico and Agua Fresca Formations, as indicated by rarefaction analyses (Figure 3). This disparity may be partly explained by taphonomic processes. Carrillo-Berumen et al. [34] reported that Chorrillo Chico and Agua Fresca exhibit high richness and abundance of sporomorphs associated with fluvio-deltaic systems (suggesting proximity to a continental source), whereas the consistent presence of *Impagidinium* dinocysts indicates deposition in a distal marine setting under hyperpycnal conditions. Such depositional differences could affect both preservation and the effective area sampled by palynological assemblages, thereby influencing apparent richness. Additionally, potential age differences among the Formations may also contribute to the observed offset, because even modest temporal differences within the Paleocene–Eocene interval could capture distinct climatic or ecological phases.

The high palynofloral diversity documented here for the LMF contrasts with previous reports [31,32] not only in the number of taxa and the absence of *Nothofagus* in the pollen rain, but also in the low similarity between studies (see Figure S2, Supporting Information). This discrepancy has been attributed to the presence of two distinct microfloras, an older assemblage lacking *Nothofagus* and a younger one in which *Nothofagus* is present, deposited under changing climatic conditions [26]. Alternatively, the observed differences may reflect variations in sampling effort or taphonomic biases rather than climate [27]. The presence of *Nothofagus* in the macroflora of the LMF supports this latter explanation [28].

Recently, Quattrocchio et al. [35] floristically linked the LMF with the nearby Laguna Manantiales Strata and related both with the Eocene Patagonian fossil floras, including Río Turbio Formation [8,33] and La Marcelina [36]. They classified LMF as a Gondwanan Subtropical flora, which exhibits a mixture of taxa with present-day Neotropical, Pantropical, and Australasian distributions, along with a low proportion of Antarctic elements [18,21]. This flora thrived in Patagonia during the early Eocene under subtropical conditions, with year-round precipitation [37,38,39]. From a taphonomic perspective, sporomorph assemblages in fluvial settings are commonly supplied by surface runoff, complemented by direct atmospheric deposition; therefore, comparisons among palynological assemblages derived from broadly similar fluvial depositional contexts should be comparable in their transport pathways and source-area integration, strengthening regional diversity and floristic comparisons. Accordingly, rarefaction curves are interpreted as standardized recorded palynological richness rather than direct estimates of true standing diversity, because transport, preservation, and depositional setting can bias taxonomic representation.

Eocene climate model simulations suggest that the LMF fell within a humid subtropical climate (Köppen–Geiger Cfa) under both 3 × CO_2_ and 6 × CO_2_ scenarios, characterized by the absence of a dry season and hot summers, with the warmest month exceeding 22 °C. Under the 3 × CO_2_ scenario, the Cfa climate extended from approximately 40° S to 50–55° S, where it transitioned poleward into the temperate oceanic/subtropical highland climate (Cfb; Figure 3c). The Cfb climate is characterized by a coldest month averaging above 0 °C, all months with average temperatures below 22 °C, and no significant seasonal differences in precipitation. During the Eocene under 3 × CO_2_ scenario, Cfb conditions dominated the southernmost latitudes of South America and the Antarctic Peninsula (Figure 3c). At present, the Cfa climate occurs in northeastern Argentina and southern Brazil, while the Cfb climate is found in southern Chile and western Argentina south of 38° S; in the northeastern Andes north of ~18° S; in southern Brazil at ~23–29° S; and along the eastern margin of Argentina at ~36–38° S [40]. This disjunct distribution of Cfb is due to the presence of extensive arid climates (Köppen’s B type) that cross the Andes along the Arid Diagonal of South America [41]. Notably, the Cfa climate would have covered the entire region south of ~40° S during the Eocene under the 6 × CO_2_ scenario (Figure 4c).

According to our hypothesis, humid temperate climates without a dry season (Köppen–Geiger Cf, within the broader mesothermal C group) are suitable for Gondwanan lineages [28]. Our niche modeling based on modern occurrence records (Figure 1) and Eocene projections under 3 × CO_2_ and 6 × CO_2_ scenarios (Figure 6) indicates a high probability of *Nothofagus* occurrence within the region influenced by the humid Southern Hemisphere westerlies, although there are differences between scenarios. Under the 3 × CO_2_ scenario, suitable conditions for *Nothofagus* were largely restricted to southwestern South America (south of ~40° S), western Australia, and New Zealand, whereas under the 6 × CO_2_ scenario, suitability expanded into Antarctica; however, projections across the continent were discontinuous and largely confined to scattered, isolated patches (Figure 6b).

Additional differences and similarities emerged when fossil occurrences were used to reconstruct the Eocene niche of *Nothofagus*. As in the projections based on modern records, suitability under both the 3 × CO_2_ and 6 × CO_2_ scenarios is concentrated within the westerlies-influenced belt south of ~40° S, encompassing southern South America, southern Australia, and New Zealand. However, under the 6 × CO_2_ scenario, areas of high suitability were largely absent (Figure 7). Consistent with expectations, the Antarctic Peninsula appears to have provided suitable conditions for *Nothofagus* during the Eocene. Importantly, the taxa is also documented by macrofossils from the Antarctic Peninsula into the early Miocene [42], indicating long-term persistence and highlighting Antarctica’s key role in the evolutionary history of the genus. The southern South America–Antarctic Peninsula region has been proposed as a source area for the migration of *Nothofagus* (and other Gondwana lineages) to Australia since the latest Cretaceous–early Cenozoic times, and dispersal routes must have involved Antarctica [43].

Our Eocene niche models for *Nothofagus* indicate low climatic suitability across much of Antarctica, suggesting that a trans-Antarctic route would have been challenging and that connectivity may have been spatially restricted and/or intermittent (Figure 7 and Figure 8). In contrast, high suitability in New Zealand and Tasmania (Figure 7) points to these regions as potential areas of persistence and diversification within eastern Gondwana, consistent with scenarios involving rare long-distance dispersal from the southern South America–Antarctic Peninsula region. Such a mechanism could help explain the Eocene divergence between the *Nothofagus* subgenus *Nothofagus* (currently restricted to South America) and *Brassospora* (restricted to Papua New Guinea and New Caledonia), dated at ~42.2 Ma (56.4–31.5 Ma; [44]). Additional support for an Antarctic component in this history comes from newly reported *Nothofagus* fossil evidence interpreted as affiliated with the Brassospora lineage from Early Eocene deposits of the La Meseta Formation, Antarctica [45].

Interpreting the mismatch between the modern-to-Eocene transfer models (Experiment 1) and the fossil-calibrated projections (Experiment 2) requires distinguishing climatic suitability from realized occupancy and considering whether climatic niches were conserved through time [46,47]. Our phylogenetic analyses support climatic niche conservatism in *Nothofagus*, but with pronounced lineage-level differentiation, such that Eocene occurrences occupy warmer climatic space closer to that of extant *Brassospora* than to the modern South American lineages that dominate the present-day realized niche [28]. Consequently, projections based on modern South American occurrences may underrepresent suitability in warmer Eocene environments, whereas fossil calibrated models better capture the climatic niche expressed by Eocene populations/lineages. This lineage structure interacts with geography: Köppen–Geiger reconstructions indicate that Antarctica was dominated during the Early Eocene by continental (D) climates (including Dsa and colder variants), with humid temperate Cf climates were spatially restricted (see Figure S3, Supporting Information), consistent with fragmented Antarctic suitability under 6 × CO_2_ (and largely absent under 3 × CO_2_) rather than a continuous corridor (Figure 6 and Figure 7). Finally, limited effective dispersal and colonization lags could have prevented continuous occupancy even where suitability was locally high [48,49], reinforcing the inference of restricted and intermittent connectivity across Antarctica during peak hothouse conditions.

An alternative, non-exclusive interpretation is that *Nothofagus* occupied only a limited subset of Antarctic environments during the Early Eocene, potentially cooler inland settings and/or higher elevations, rather than being widespread across the continent [7,9,50]. This view is consistent with the offshore early Eocene (53.9–51.9 Ma) pollen record from Wilkes Land (Site U1356; Figure S4, Supporting Information), which indicates mesothermal, stratified forests including Bombacoideae, Strasburgeria, palms, and Proteaceae, and implies strong spatial climatic gradients that could have restricted temperate rainforest elements to cooler refugial areas [7,9]. Notably, Wilkes Land and the LMF exhibit comparable diversity under equivalent sampling effort (Figure S5, Supporting Information) and similar mesothermal reconstructions (e.g., MAT = 16 ± 3 °C; AP = 132 ± 55 cm) for Wilkes Land [9]. However, our niche models (Figure 6 and Figure 7) suggest that many inland and/or higher-elevation Antarctic settings remained of low suitability for *Nothofagus* during this hothouse interval, whereas suitability along Antarctica was discontinuous and largely confined to discrete coastal sectors. Under 6 × CO_2_ scenario, both experiments recover a coastal window of suitability in the Wilkes Land sector (Figure 6b and Figure 7b; Figure S4, Supporting Information).

Beyond Antarctica, our projections also identify suitable habitats in Australia, including portions of the hinterland and selected coastal regions (Figure 6 and Figure 7), consistent with earlier paleobotanical syntheses [50]. The apparent scarcity or absence of *Nothofagus* evidence in some of these climatically suitable Australian regions during the hothouse interval may therefore reflect dispersal limitation, whereby taxa fail to occupy environmentally suitable areas because propagules do not reach them [51]. Targeted discovery of additional *Nothofagus* megafossils (e.g., leaves, wood, or reproductive structures) from Australia or East Antarctica would provide critical constraints on whether these regions hosted undocumented populations and would help discriminate among competing scenarios of connectivity, range fragmentation, and dispersal limitation during the Early Eocene.

In South America, our niche modeling indicates that both Cfa and Cfb climates were suitable for *Nothofagus* during the Eocene (Figure 3 and Figure 4). However, according to the fossil record, floras north of LMF (Figure 8), such as those at Laguna del Hunco, lack megafossils of *Nothofagus*. This pattern is also observed in other Eocene floras, such as Río Pichileufú and Lota-Coronel [15,52]. These have historically been classified as a distinct floristic unit known as Mixed Floras without *Nothofagus* [15,22,23,53]. It is not clear why *Nothofagus* doesn’t reach lower latitudes, and this may be attributed to rapid climatic or landscape changes occurring over short timescales [54]. The absence of *Nothofagus* in the Laguna del Hunco flora, dated at 51.91 ± 0.22 Ma during the Early Eocene Climatic Optimum, may be explained by climatic differences relative to the slightly older Ligorio Márquez flora (~53 Ma). However, the absence of this taxon in the Pampa Jones flora, located approximately 1.4° north of Laguna del Hunco and dated at 54.24 ± 0.45/53.64 ± 0.35 Ma [54], suggests that regional climatic differences, rather than temporal ones, may account for its distribution.

Figure 8 compares the mean sea-level pressure during the austral summer (Figure 8a) and winter (Figure 8b). The fossil floras located north of LMF were under the influence of an anticyclonic system during the summer, which likely acted as an environmental filter, limiting the northward distribution of *Nothofagus* during the Eocene. Hinojosa et al. [28] evaluated the phylogenetic signal of environmental variables related to temperature and precipitation. Their findings indicate that *Nothofagus* is highly sensitive to precipitation changes, consistent with a stabilizing selection model within an Ornstein-Uhlenbeck evolutionary framework. As a result, the annual variation in anticyclonic influence at mid-latitudes in South America likely acted as an effective environmental filter for *Nothofagus* during the Eocene and as a stabilizing selection force throughout the Cenozoic. The modern distribution of *Nothofagus* in Mediterranean climates in South America supports the hypothesis of stabilizing selection by the annual precipitation regime influencing the genus distribution in this region. A plausible trait-based mechanism underlying precipitation-linked filtering in *Nothofagus* is recruitment limitation under seasonal water deficit, as manipulative experiments show that seedling survival, growth, and eco-physiological performance are strongly constrained by water shortage and drought associated with warming [55,56].

The Eocene Cfa mesothermal climate, occurring at mid-latitudes in South America, has been proposed as a source of diversity for tropical regions [28]. Several taxa exhibit a disjunct distribution between temperate and tropical latitudes today, reflecting ancestral mesothermal climatic niches that shifted from high or mid-latitudes toward lower latitudes due to climatic and tectonic changes [28,41,57,58,59,60]. This suggests that modern areas under Cfa mesothermal climates in South America may harbor ancient lineages rooted in the early Cenozoic paleofloras of southern South America, as supported by molecular evidence [61].

To explore the paleoclimatic context of our fossil assemblages, we employed multiple general circulation models (GCMs) simulating conditions for the Hothouse interval [1,29]. These models provide spatially explicit reconstructions of key climate variables and allow for comparisons across regions, even in areas with limited fossil data. However, their coarse resolution and reliance on uncertain boundary conditions present limitations, particularly for local-scale interpretations [62]. By using an ensemble of models, we aim to capture a range of plausible scenarios, while emphasizing that model-based interpretations must be integrated with independent geological and paleontological evidence, as done in this study.

## 4. Materials and Methods

### 4.1. Rarefied Richness Across Eocene Hothouse Assemblages

To compare the diversity during the hothouse period in southern South America between Ligorio Marquez and other floras, we built a dataset with the abundance of morphospecies from Ligorio Marquez Formation (LMF), Laguna del Hunco (LH), Chorrillo Chico Formation (CCF), Agua Fresca Formation (AF), Lower Rio Turbio (LRT), and Upper Rio Turbio (URT).

The Ligorio Márquez Formation (LMF) crops out in Chilean Patagonia and preserves fossil-bearing continental deposits that record early Paleogene environments in the region. The age of the LMF is constrained by radiometric dating and palynological evidence. U–Pb zircon ages from stratigraphically ascending levels (57.3 ± 2.7 Ma, 53.7 ± 2.9 Ma, and 50.5 ± 2.5 Ma) provide maximum depositional ages spanning the late Paleocene (Thanetian) to Early Eocene (Ypresian), and constrain the upper part of the Formation to the Early Eocene [63]. These ages are consistent with K–Ar and ^40^Ar/^39^Ar dates reported for the overlying Basaltos Inferiores Meseta Chile Chico (BIMCC) unit [27,64]. Taken together, the available geochronological constraints indicate that the fossiliferous interval yielding the LMF flora most plausibly falls within the Early Eocene (Ypresian).

The age of the flora has been approached by different authors. Suárez et al. [26] and Troncoso et al. [31] reported 19 leaf taxa, mainly associated with Lauraceae, and 12 spore and pollen taxa, highlighting the absence of *Nothofagus*. They compared their findings with the Chilean fossil flora of Lota-Coronel [52] and suggested a similar age. The Lota-Coronal flora is deposited in the coal-bearing strata of the Curanilahue Formation in Central Chile and has been assigned to the Upper Paleocene-Early Eocene (but see [65]). In addition, the occurrence of *Malvacipollis diversus* and *Retitricolporites medius* supports an early Eocene age for the LMF flora [28,35,66] (see Supplementary Information for discussion).

The LMF data were collected from 35 horizons and sampled in 50 m sections. From the base to the top, 23 samples were taken from deposits of floodplain and sandstones (located in the first 20 m of the section), eight samples from coal lines and siltstones (located between 20 and 40 m), and the remaining four samples from sandstones at the top section (between 40 and 50 m, see Figure S6 Supporting Information). The palynological samples were prepared by the standard procedure of digesting the sample in HF and HCl acids, separating organic matter by heavy liquids, and oxidizing [67]. A complete oxidized slide per sample was scanned with a 20X Zeiss (Oberkochen, Germany) plan apochromatic objective, and 300 palynomorphs per slide were counted when possible [66]. The age of LMF is Early Eocene [28,63,64,66].

The LH microflora was reported by Barreda et al. [68]. The samples were collected from seven stratigraphic levels in the Huitrera Formation, in Chubut, Argentina. These outcrops are the remains of a fossil caldera lake in the Middle Chubut River. 56 spore and pollen species and 28 plant families were recovered. The age of LH is Early Eocene [14,16].

The Chorrillo Chico Formation and Agua Fresca Formation microflora were reported by Carrillo-Berumen et al. [34]. These Formations are exposed in a Paleogene section of Punta Prat, Chile, in the southern Magallanes-Austral Basin. Both units contain a palynological assemblage composed of marine and continental palynomorphs, indicative of a marine depositional environment with terrigenous input. Chorrillo Chico has 56 pollen and spore species, with 16 angiosperms, 12 gymnosperms, and 28 spores. The age of CC is Paleocene (Thanetian). Agua Fresca has 50 pollen and spore species, with 14 angiosperms, 12 gymnosperms, and 24 spores. The age of AF is Early Eocene [69].

The Rio Turbio Formation microflora was reported by Fernandez et al. [8]. Samples collected from the Lower section of the Formation have 108 pollen and spore species, with 68 Angiosperm, 10 Gymnosperm, and 30 spores. The Upper section has 101 pollen and spore species, with 67 Angiosperm, 8 Gymnosperm, and 26 spores. The age of RTF is constrained as the mid-late Eocene (~47–34 Myr [70]).

We evaluated differences in species richness among fossil microfloral assemblages using rarefaction/extrapolation [71] curves in iNEXT [72]. For formal inference, we compared rarefied species richness (q = 0) at a common number of individuals, m_0_, defined as the minimum observed total abundance among assemblages retained for comparison (thus avoiding extrapolation). Assemblages with very low total abundance were excluded using an explicit threshold rule, N < max [1000, 0.5 × N2nd_smallest_], where N2nd_smallest_ is the second-smallest observed sample size across assemblages. All pairwise tests were then conducted among the remaining sites using the same m_0_. For each assemblage, we generated 200 bootstrap replicates via multinomial resampling (total N fixed), reran iNEXT for each replicate, and extracted S_X_(m_0_) (or the nearest reported sample size). Pairwise differences were computed as Δ = *S_A_* (*m*_0_) − *S_B_* (*m*_0_), where *S_X_* (*m*_0_) is the rarefied richness of assemblage X at m_0_; Δ > 0 indicates higher richness in A. We report bootstrap 95% confidence intervals and two-sided bootstrap *p*-values, with Bonferroni correction for multiple comparisons.

### 4.2. Mesothermal, Humid Conditions at LMF in 3× and 6× pCO_2_ DeepMIP Simulations

To assess the mesothermal paleoclimate accuracy of the LMF paleoclimate, previously published by Hinojosa et al. [28], we compared it with the estimate obtained by the Deep-Time Model Intercomparison Project (DeepMIP, Lunt et al. [29]). We used simulations of preindustrial control and Early Eocene simulations at CO_2_ concentrations of approximately 800 ppm (×3 pre-industrial levels) in seven models and 1600 ppm (×6 pre-industrial levels) in three models (Table 6). DeepMIP Eocene experiments are designed to represent Early Eocene boundary conditions rather than a single discrete time slice; therefore, we use 3 × CO_2_ and 6 × CO_2_ as bracketing forcings that span plausible atmospheric CO_2_ levels for the LMF age range constrained by U–Pb maximum depositional ages (~54–53 Ma) and for comparison with floras close to peak EECO conditions. Accordingly, 3 × CO_2_ provides a conservative baseline, whereas 6 × CO_2_ approximates peak hothouse forcing. According to Lunt et al. [29], the paleogeography, vegetation, and river routing for the Eocene simulations correspond to those of Herold et al. [73]. The solar constant, orbital configuration, and non-CO2 greenhouse gas concentrations were set to preindustrial values. Soil properties were set to homogeneous global mean values derived from the preindustrial simulation, and there were no continental ice sheets in the Eocene simulations. For details on each model, please refer to Lunt and collaborators [29,74].

From each Early Eocene model in Table 6, we obtained 22 raster environmental variables related to temperature, precipitation, evapotranspiration, hydric balance (evapotranspiration minus precipitation), and orography. Because the DeepMIP models have varying native spatial resolutions, all fields were harmonized by reprojection onto a common 5′ × 5′ global grid using the coordinate reference system ‘+proj = longlat + datum = WGS84’ (extent: −180° to 180° longitude and −90° to 90° latitude). Values on the target grid were computed using the ‘bilinear’ interpolation method from the Terra R package. We emphasize that this procedure is a grid-matching interpolation for comparability and mapping, rather than a physical (dynamical or statistical) downscaling; therefore, it does not generate new fine-scale climate information or resolve microclimates or local orographic effects in complex topography. Accordingly, site-level values should be interpreted as regional approximations. A total of 22 raster-based environmental variables were obtained, including data on temperature, precipitation, evapotranspiration, hydric balance (evapotranspiration minus precipitation), and orographic conditions during the Eocene (Table 7). The paleo-coordinates of the LMF were plotted onto these rasters, and values for each environmental variable were extracted using the ‘extract’ function of the terra R package [77]. Finally, an ensemble-mean model was calculated for both the 3 × CO_2_ and 6 × CO_2_ experiments. Using reconstructed monthly paleoclimate rasters of temperature and precipitation, we classified the Early Eocene climate of South America into Köppen–Geiger climate types [40], and the LMF, along with other Eocene floras, was classified accordingly.

### 4.3. Nothofagus Ecological Niche Models and Early Eocene Suitability Across Gondwana

We performed ecological niche modeling [78] to estimate the potential distribution of *Nothofagus* during the Early Eocene under two complementary experiments. In Experiment 1 (modern-to-Eocene transfer), models were calibrated using the modern distribution of *Nothofagus* under pre-industrial (PI) climatic conditions and subsequently projected onto Early Eocene climates across Gondwana (South America, Antarctica, Australia, and New Zealand) under both 3 × CO_2_ and 6 × CO_2_ scenarios. Modern occurrences were spatially thinned prior to modeling, and models were fitted separately for each GCM listed in Table 6 using the corresponding environmental predictors (Table 7). For each GCM, we defined the calibration region as a 450 km buffer around the thinned occurrences, masked the PI environmental stack to this domain, and sampled 15,000 random background points within it. We selected 450 km as a pragmatic extent to approximate the accessible area (M) for background sampling and to ensure that the calibration domain encompassed the main geographic components of the native *Nothofagus* range across Gondwana, including New Zealand, New Caledonia, and Papua–New Guinea, thereby avoiding artificial truncation of the accessible environmental background. Predictor redundancy was reduced by removing near-zero-variance variables and filtering multicollinearity using Spearman correlations computed from 10,000 randomly sampled raster cells; correlated predictors were removed using a threshold of r ≥ 0.80. Model complexity was tuned using k-fold cross-validation and a grid search over feature-class combinations and regularization multipliers (1–3, step 0.5), selecting the configuration that maximized AUC and minimized the omission rate on holdout test presences at the 10th-percentile training presence threshold (OR_P10), and favoring simpler feature sets when performance differences were negligible. Final models were refitted with the selected settings and projected using cloglog output.

In Experiment 2 (Eocene-to-Eocene calibration), we reconstructed the Eocene niche directly from fossil evidence by calibrating models with Eocene *Nothofagus* occurrences (in paleocoordinates) and projecting within the Early Eocene climatic space for each CO_2_ scenario and GCM (Table 1). Because fossil occurrences are geographically restricted, we defined the calibration region using a fixed bounding box enclosing the fossil localities, cropped the Early Eocene predictor stack to this region, and sampled 15,000 random background points within the cropped domain. The same workflow for predictor screening (near-zero-variance removal and Spearman correlation filtering at r ≥ 0.80), hyperparameter tuning (cross-validation and grid search using AUC, we also report OR_P10 evaluated on all Eocene presences (*n* = 11), which yields discrete values (multiples of 1/11)), and prediction settings (cloglog output) was applied. To quantify uncertainty in both experiments, we implemented 10 bootstrap replicates per GCM and scenario by resampling presences with replacement while keeping background points constant, and summarized replicate projections as ensemble-mean rasters. Models were fitted under the maximum-entropy framework [79,80] using SDMtune [81], with models trained via the Maxnet method implemented in the R package maxnet [82].

To summarize high-suitability areas from these presence-only models, we used the 10th percentile training presence threshold (P10) as a reference for visualizing the continuous cloglog suitability surfaces in Experiments 1 and 2 (i.e., color scales were set to begin near P10 to emphasize the suitability range supported by training presences). P10 is a commonly used MaxEnt/Maxnet threshold that is comparatively robust to a small number of low-suitability outlier occurrences and provides a pragmatic compromise between omission error and overprediction when interpreting suitability surfaces [83,84]. Because P10 values differed between the two modeling settings, we computed experiment-specific P10 thresholds for each GCM and CO_2_ scenario, using the mean P10 across bootstrap replicates.

Finally, we used the bioclimatic thermal regime proposed by Nix [4]: megathermal climate (MAT ≥ 22 °C, MAP > 549 mm); mesothermal climate (MAT ≥ 14–22 °C, MAP > 549 mm); and microthermal climate (MAT < 14 °C, MAP 719–3000 mm) and Köppen -Geiger climate classification [40]. This Classification defines five major climate types based on temperature and precipitation thresholds and was implemented in R using a translation of the Beck et al. [85] algorithm.

## 5. Conclusions

The Ligorio Márquez Formation hosted a highly diverse palynoflora comparable to other diversity-rich Eocene assemblages in southern South America, and it developed under mesothermal, humid Cf conditions.

Niche-model projections indicate that climatically suitable habitats for *Nothofagus* were broadly concentrated in western Gondwana (southern South America, the Antarctic Peninsula, Australia, and New Zealand). In contrast, suitability across Antarctica was discontinuous, suggesting a limited role for Antarctica as a continent-wide dispersal corridor during peak hothouse conditions; an enhanced South Pacific High likely acted as an additional environmental filter shaping South American distributions.

The Eocene prevalence of Cf-like climates implies that modern regions with comparable conditions may retain lineages with deep Cenozoic roots, while multi-model paleoclimate simulations provide a powerful interpretive framework that should be corroborated with independent geological and fossil evidence.

## Figures and Tables

**Table 1:** Pairwise comparisons of rarefied species richness (q = 0) among microfloral assemblages at a common sample size (m_0_ = 2007 individuals). Δ = SA(m_0_) − SB(m_0_), where SX(m_0_) is the rarefied richness of assemblage X at m_0_. Uncertainty corresponds to the bootstrap 95% confidence interval (CI95). Two-sided *p*-values are based on the bootstrap distribution of Δ(*p*), and *p*_Bonf indicates *p*-values adjusted for multiple pairwise tests using the Bonferroni procedure. Microflora compared: Ligorio Marquez Formation (LMF); Chorrillo Chico (CC); Agua Fresca (AF); Rio Turbio Formation lower and upper (RTlow and RTupp).

Comparison	m0	Δ	CI95	*p*	*p*_Bonf
LMF. vs. CC	2007	28.6	[22.8, 34.3]	<0.005	<0.005
LMF vs. AF	2007	33.6	[28.5, 38.0]	<0.005	<0.005
RTinf vs. CC	2007	33.7	[29.6, 37.8]	<0.005	<0.005
RTinf vs. AF	2007	38.7	[35.3, 42.4]	<0.005	<0.005
CC vs. RTsup	2007	−32.4	[−36.5, −28.0]	<0.005	<0.005
AF vs. RTsup	2007	−37.4	[−41.3, −33.5]	<0.005	<0.005
CC vs. AF	2007	5.0	[0.8, 9.3]	0.01	0.1
LMF vs. RTinf	2007	−5.1	[−10.7, −0.3]	0.04	0.4
LMF vs. RTsup	2007	−3.8	[−9.6, 0.7]	0.13	1
RTinf vs. RTsup	2007	1.4	[−2.5, 5.2]	0.47	1

**Table 2:** Model configuration and predictive performance across general circulation models (GCMs) and CO_2_ scenarios for experiment 1. For each GCM and scenario, we report the selected Maxnet feature-class set (fc), the regularization multiplier (reg), predictive performance on the holdout test data expressed as the area under the ROC curve (AUC_test), and the omission rate on the holdout test presences at the 10th percentile training presence threshold (OR_P10). Feature classes are abbreviated as follows: lh (linear + hinge), lqh (linear + quadratic + hinge), and lqph (linear + quadratic + product + hinge). Models calibrated under 3 × CO_2_ were projected to the Early Eocene under 3 × CO_2_ for all GCMs, whereas CESM, GFDL, and INMCM were additionally used for 6 × CO_2_ projections.

Model	Scenario	fc	reg	AUC_Test	OR P10
CESM	3 × CO_2_	lh	1	0.88	0.105
COSMOS	3 × CO_2_	lh	1	0.88	0.110
GFDL	3 × CO_2_	lh	1	0.86	0.099
HADCM3B	3 × CO_2_	lqh	1	0.88	0.128
HADCM3BL	3 × CO_2_	lqh	1	0.88	0.126
IPSL	3 × CO_2_	lh	1	0.88	0.113
MIROC	3 × CO_2_	lqh	1	0.87	0.118
CESM	6 × CO_2_	lh	1	0.88	0.105
GFDL	6 × CO_2_	lh	1	0.86	0.099
INMCM	6 × CO_2_	lqph	1	0.87	0.110

**Table 3:** Top three predictors (permutation importance) for Experiment 1 (modern-to-Eocene transfer) across GCMs and CO_2_ scenarios. Ranked predictors correspond to the three variables listed in Table 7 with the highest permutation importance in Maxnet models trained with modern *Nothofagus* occurrences under pre-industrial conditions and projected to Early Eocene climates. Values in parentheses indicate permutation importance within each model run.

Climate Model	Scenario	Rank 1	Rank 2	Rank 3
CESM	3 × CO_2_	Var6 (34)	Var4 (21)	Var18 (12)
COSMOS	3 × CO_2_	Var15 (29)	Var2 (24)	Var12 (16)
GFDL	3 × CO_2_	Var15 (40)	Var14 (38)	Var12 (18)
HADCM3B	3 × CO_2_	Var1 (36)	Var6 (33)	Var10 (22)
HADCM3BL	3 × CO_2_	Var6 (41)	Var2 (28)	Var21 (12)
IPSL	3 × CO_2_	Var9 (33)	Var6 (31)	Var22 (18)
MIROC	3 × CO_2_	Var4 (28)	Var14 (23)	Var12 (18)
CESM	6 × CO_2_	Var6 (34)	Var4 (21)	Var18 (12)
GFDL	6 × CO_2_	Var15 (40)	Var14 (38)	Var12 (18)
INMCM	6 × CO_2_	Var4 (45)	Var12 (22)	Var13 (21)

**Table 4:** Model configuration and predictive performance across general circulation models (GCMs) and CO_2_ scenarios for experiment 2. For each GCM and scenario, we report the selected Maxnet feature-class set (fc), the regularization multiplier (reg), and predictive performance on the holdout test data expressed as the area under the ROC curve (AUC_test). We also report the omission rate at the 10th percentile training presence threshold (OR_P10), evaluated on all available Eocene presence records (*n* = 11); because of the small sample size, omission rates are necessarily discrete (multiples of 1/11). Feature classes are abbreviated as follows: lh (linear + hinge), lqh (linear + quadratic + hinge), and lqph (linear + quadratic + product + hinge). Models calibrated under 3 × CO_2_ were projected to the Early Eocene under 3 × CO_2_ for all GCMs, whereas CESM, GFDL, and INMCM were additionally used for 6 × CO_2_ projections.

Model	Scenario	fc	reg	AUC_Test	OR_P10
CESM	3 × CO_2_	lh	1	0.91	0.182
COSMOS	3 × CO_2_	l	2.5	0.93	0.182
GFDL	3 × CO_2_	lq	3	0.98	0.091
HADCM3B	3 × CO_2_	lqph	1	0.92	0.182
HADCM3BL	3 × CO_2_	lqph	1	0.93	0.182
IPSL	3 × CO_2_	lh	1	0.89	0.182
MIROC	3 × CO_2_	lq	1	0.95	0.182
CESM	6 × CO_2_	lh	1	0.91	0.182
GFDL	6 × CO_2_	lq	3	0.98	0.091
INMCM	6 × CO_2_	lq	1.5	0.87	0.182

**Table 5:** Top three predictors (permutation importance) for Experiment 2 (Eocene-to-Eocene calibration) across GCMs and CO_2_ scenarios. Ranked predictors correspond to the three variables listed in Table 2 with the highest permutation importance in Maxnet models trained with modern *Nothofagus* occurrences under pre-industrial conditions and projected to Early Eocene climates. Values in parentheses indicate permutation importance within each model run.

Climate Model	Scenario	Rank 1	Rank 2	Rank 3
CESM	3 × CO_2_	Var4 (70)	Var14 (8)	Var22 (7)
COSMOS	3 × CO_2_	Var4 (72)	Var14 (11)	Var8 (9)
GFDL	3 × CO_2_	Var15 (45)	Var14 (22)	Var21 (21)
HADCM3B	3 × CO_2_	Var14 (48)	Var1 (21)	Var9 (12)
HADCM3BL	3 × CO_2_	Var14 (38)	Var4 (20)	Var8 (12)
IPSL	3 × CO_2_	Var6 (61)	Var19 (7)	Var13 (7)
MIROC	3 × CO_2_	Var14 (33)	Var2 (22)	Var3 (20)
CESM	6 × CO_2_	Var4 (70)	Var14 (8)	Var22 (7)
GFDL	6 × CO_2_	Var15 (45)	Var14 (22)	Var21 (21)
INMCM	6 × CO_2_	Var2 (45)	Var3 (22)	Var21 (21)

**Table 6:** Global circulation models from the Deep-Time Model Intercomparison Project (DeepMIP-Eocene, Lunt, et al. [29]).

Model	Short Name	CO_2_ Experiment	Simulation Reference
CESM1.2_CAM5	CESM	×3, ×6	[75]
COSMOS-landveg_r2413	COSMOS	×3	[29]
GFDL_CM2.1	GFDL	×3, ×6	[29]
HadCM3B_M2.1aN	HadCM3	×3	[29]
HadCM3B_L M2.1aN	HadCM3	×3	[29]
INM-CM4-8	INMCM	×6	[29]
IPSLCM5A2	IPSL	×3	[76]
MIROC4m	MIROC	×3	[29]

**Table 7:** Twenty-two environmental variables derived from the ensemble mean model used for niche modeling.

Variable Name	Variable
Var1	Mean annual temperature (°K)
Var2	Range Temperature (temp max − temp min) × 10 (°K)
Var3	JJA mean temp (°K)
Var4	DJF mean temp (°K)
Var5	Annual precipitation (mm)
Var6	JJA precipitation (mm)
Var7	DJF precipitation (mm)
Var8	Annual evaporation (mm)
Var9	JJA evaporation (mm)
Var10	DJF evaporation (mm)
Var11	Annual Hydric Balance (EP, Bio8-Bio5) (mm)
Var12	JJA EP (mm)
Var13	DJF EP (mm)
Var14	Orography (m.a.s.l)
Var15	Temperature of the warmest month (°K)
Var16	Temperature of the coolest month (°K)
Var17	Precipitation of the wetter month (mm)
Var18	Precipitation of the driest month (mm)
Var19	Evaporation of the warmest month (mm)
Var20	Evaporation of the coolest month (mm)
Var21	EP max (Var19-Var17) (mm)
Var22	EP min (Var20-Var18) (mm)

## Data Availability

The original contributions presented in this study are included in the article/Supplementary Material. Further inquiries can be directed to the corresponding author.

## Supplementary Materials

The following supporting information can be downloaded at https://www.mdpi.com/article/10.3390/plants15071122/s1, Figure S1: Photographs illustrating the diversity of pollen and spores recovered from the Ligorio Márquez Formation, Chile. Pathfinder coordinates are provided for each specimen. Figure S2: UPGMA Cluster analysis based on Sorensen Dissimilarity between this study and previously published work by Troncoso et al. 2002 [31] and Macphail et al. 2013 [32]. Figure S3: Köppen–Geiger reconstructions for the Southern Hemisphere. (A) Ensemble mean based on seven models under 3 × CO_2_. (B) Ensemble mean based on three models under 6 × CO_2_. During the Early Eocene, Antarctica was predominantly characterized by continental (D) climates, including Dsa and colder variants, whereas humid temperate Cf climates were spatially restricted. Figure S4: Modeled suitability for Nothofagus (threshold > 0.5) across Antarctica and the Australian region under (a) 3 × CO_2_ and (b) 6 × CO_2_ for Experiment 1 (modern-to-Eocene transfer), and under (c) 3 × CO_2_ and (d) 6 × CO_2_ for Experiment 2 (Eocene-to-Eocene calibration). The yellow dot marks the Wilkes Land offshore drill site (IODP Site U1356). Contours show mean sea-level pressure (hPa). Figure S5: Rarefaction (continuous lines) and extrapolation (break lines) analysis comparing the Ligorio Marquez Formation with the Paleogene microflora of Rio Turbio, Laguna del Hunco, Agua Fresca, Chorrillo Chico, and the offshore core Wilkes Land. Shadow areas correspond to a 95% confidence interval. Figure S6: Ligorio Marquez Formation geological description and age discussion. Table S1: Microflora from the Ligorio Márquez Formation, including the pollen and spores, published by Troncoso et al., 2002 [31], and Macphail et al., 2013 [32]. Table S2: Microflora from the Ligorio Márquez Formation and their inferred botanical affinities, including the frequency of each morphotaxon used in rarefaction and extrapolation analyses. Table S3. Assemblage-level sampling effort and richness (N, S_obs, and S(m0) with 95% bootstrap CIs).
