# Supplementary material for: Early Eocene Palynofloral Diversity and Nothofagus Niche Modeling Across Western Gondwana
Source: Plants (Basel). 2026 Apr 7;15(7):1122. doi: 10.3390/plants15071122 (PMC13074919; doi:10.3390/plants15071122)
Supplement: Supplementary file 1 [file plants-15-01122-s001.zip › plants-4140483-supplementary.pdf]

Supplementary Materials

Early Eocene Palynofloral Diversity and Nothofagus Niche Modeling across Western Gondwana

L.F. Hinojosa[1*], F.M. Carvajal[2], M. Quattrocchio[3], D. Fernández[4], M.F. Pérez[5]

Figure S1: Photographs illustrating the diversity of pollen and spores recovered from the Ligorio Márquez Formation, Chile. Pathfinder coordinates are provided for each specimen:

Figure S2: UPGMA Cluster analysis based on Sorensen Dissimilarity between this study and previously published work by Troncoso et al. 2000 and Macphail et al. 2013 (papers cited in the main article)

Figure S3: Köppen–Geiger reconstructions for the Southern Hemisphere. (A) Ensemble mean based on seven models under 3×CO_2. (B) Ensemble mean based on three models under 6×CO_2. During the Early Eocene, Antarctica was predominantly characterized by continental (D) climates, including Dsa and colder variants, whereas humid temperate Cf climates were spatially restricted.

Figure S4: Modeled suitability for Nothofagus (threshold > 0.5) across Antarctica and the Australian region under (a) 3×CO_2 and (b) 6×CO_2 for Experiment 1 (modern-to-Eocene transfer), and under (c) 3×CO_2 and (d) 6×CO_2 for Experiment 2 (Eocene-to-Eocene calibration). The yellow dot marks the Wilkes Land offshore drill site (IODP Site U1356). Contours show mean sea-level pressure (hPa).

Figure S5: Rarefaction (continuous lines) and extrapolation (break lines) analysis comparing the Ligorio Marquez Formation with the Paleogene microflora of Rio Turbio, Laguna del Hunco, Agua Fresca, Chorrillo Chico, and the offshore core Wilkes Land. Shadow areas correspond to a 95% confidence interval.

Figure S6: Ligorio Marquez Formation geological description and age discussion.

Table S1: Microflora from the Ligorio Márquez Formation, including the pollen and spores, published by Troncoso et al, 2000, and Macphail et al, 2013.

Table S2: Microflora from the Ligorio Márquez Formation and their inferred botanical affinities, including the frequency of each morphotaxon used in rarefaction and extrapolation analyses.

Table S3. Assemblage-level sampling effort and richness (N, S_obs, and S(m0) with 95% bootstrap CIs)**.**

Figure S1: Photographs illustrating the diversity of pollen and spores recovered from the Ligorio Márquez Formation, Chile. Pathfinder coordinates are provided for each specimen:

PLATE 1: *Laevigatosporites ovatus* Wilson & Webster 1946, sample LMF020, EF H48. 2) *Peromonolites* sp., sample LMF023, EF S10/2. 3) *Peromonolites vellosus* Partridge in Stover & Partridge 1973, sample LMF020, EF C47/4. 4) *Polypodiisporites* sp., sample LMF034, EF H34/2. 5) *Baculatisporites comaumensis* (Cookson 1953) Potonie 1956, sample LMF051, EF E30/2. 6) *Baculatisporites turbioensis* Archangelsky 1972, sample LMF051, EF Q41/1-2. 7) *Biretisporites crassilabratus* Archangelsky 1972, sample LMF024, EF T48/4. 8) *Biretisporites* sp. II Archangelsky 1972, sample LMF025, EF K24/3. 9) *Biretisporites* cf. *crassilabratus* Archangelsky 1972, sample LMF020, EF M33/3. 10) *Cingutriletes australis* (Cookson) Archangelsky 1972, sample LMF020, EF L18/4. 11) *Biretisporites* sp., sample LMF026, EF G20/4. 12) *Concavisporites* sp., sample LMF021, EF H7/4. 13) *Cyatheacidites* cf. *annulatus* Cookson 1947, sample LMF022 EF C30/11. 14) *Corrugatisporites* cf. *argentinus* Archangelsky 1972, sample LMF021, EF R12/4. 15) *Dictyophyllidites* cf. *crenatus* Dettman 1963, sample LMF028, EF S15 4.

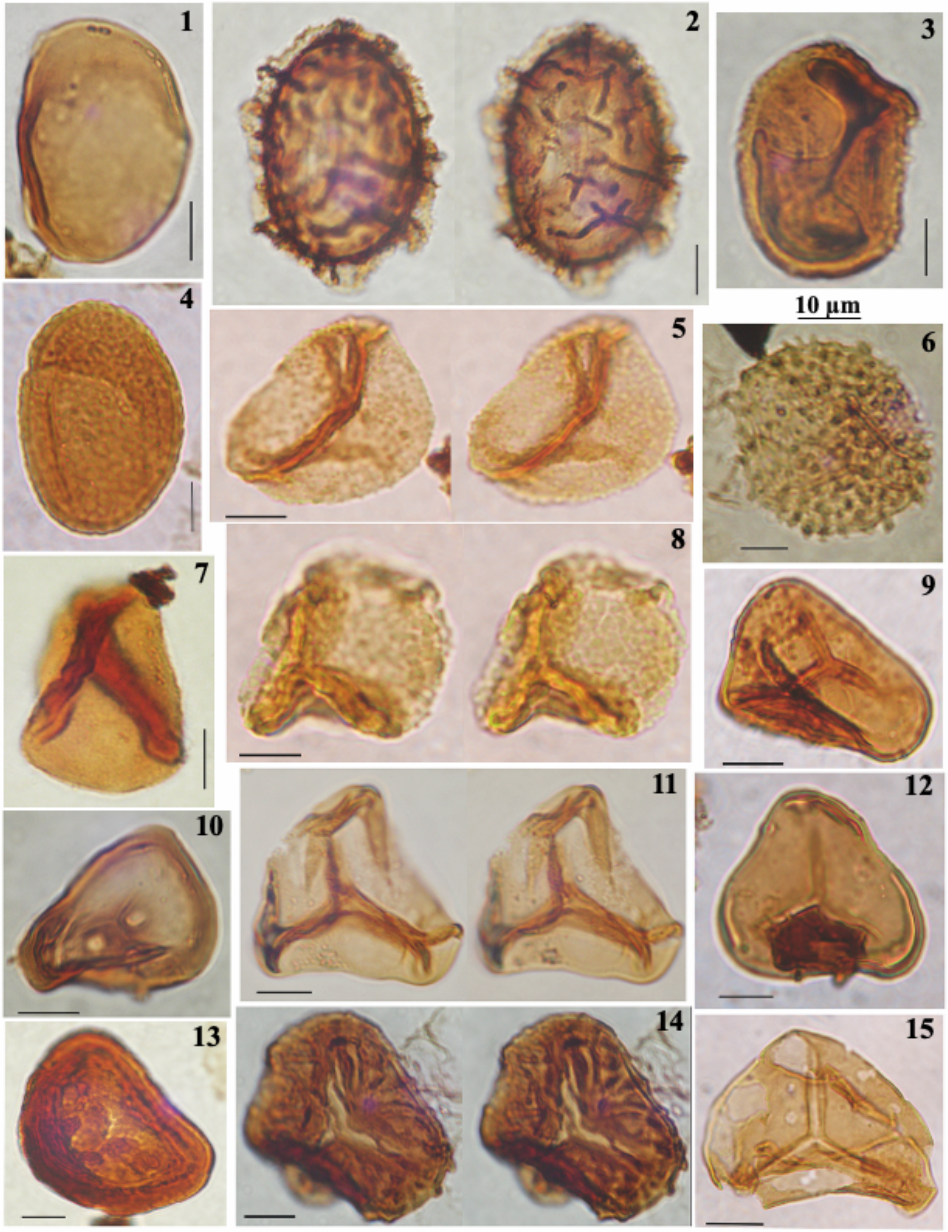

PLATE 2

1) *Deltoidospora minor* (Couper) Pocock 1970, sample LMF020, EF W48/2. 2) *Dictyophyllidites* sp., sample LMF020, EF P37 1/3. 3) *Dictyophyllidites pectinataeformis* (Bolkhovitina) Dettman 1963, sample LMF052, EF N46/1. 4) *Echinatisporis* sp., sample LMF023, EF L12/1-2. 5) *Leiotriletes regularis* (Pflug) Krutzsch 1959, sample LMF051, E39/1. 6) *Ischyosporites* cf. *crateris* Balme 1957, sample LMF025, EF V9/2. 7) *Ischyosporites* sp., sample LMF052, EF M35/4. 8) *Kluklisporites* sp., sample LMF051, EF M40. 9) *Retitriletes* sp., sample LMF 024, EF J48/1. 10) *Pseudoschizaea circula* (Wolf) Christopher, 1976, sample LMF032, EF T46/4. 11) *Verrucatitriletes* sp., sample LMF052, EF N18/1-3. 12) *Trilites* cf. *parvallatus* Krutzsch 1959, sample LMF025, EF O19/4.

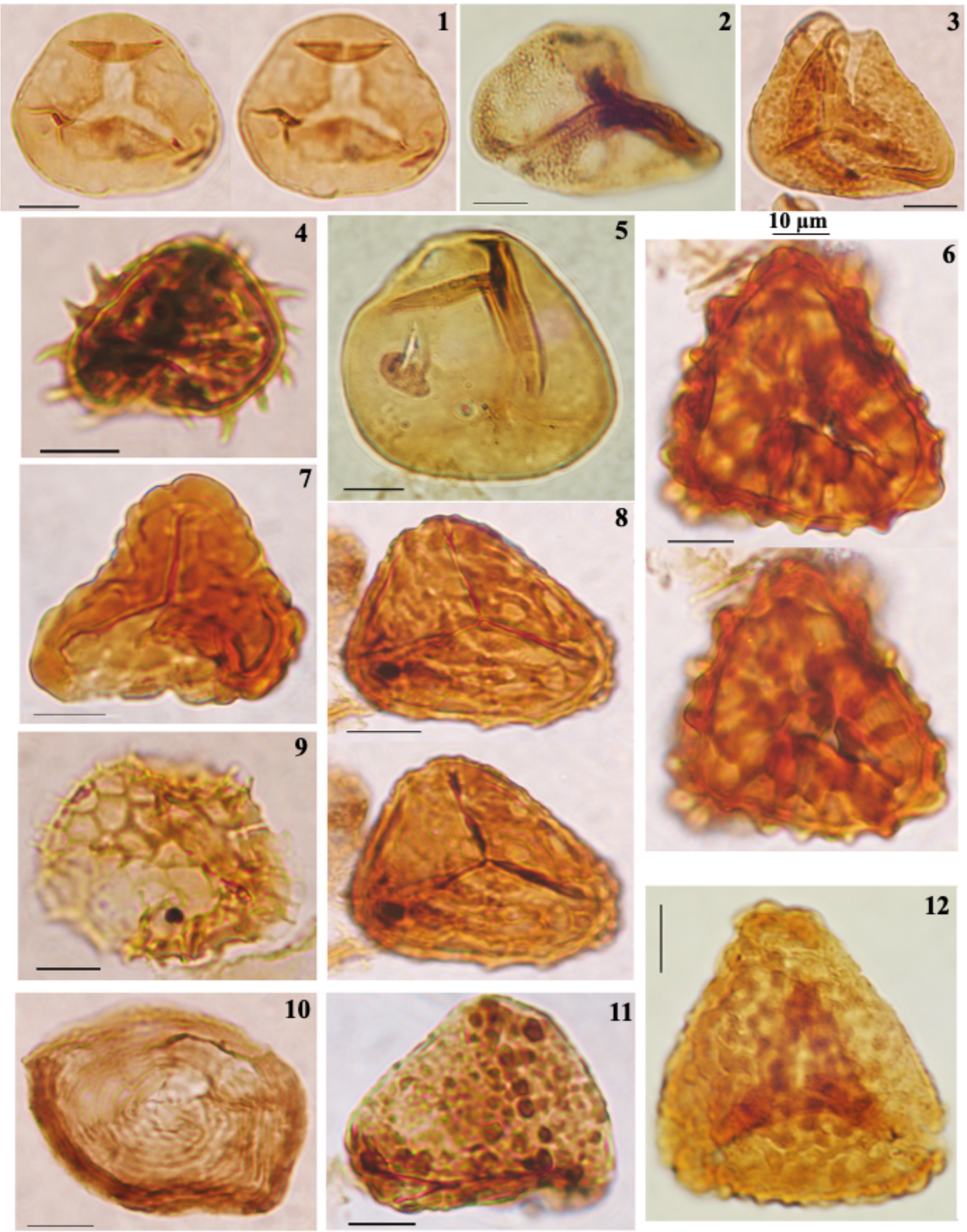

PLATE 3

1) *Araucariacites australis* Cookson, 1947, sample LMF020, EF J20/1. 2) *Dacrycarpites australiensis* Cookson & Pike 1953, sample LMF052, EF L40. 3) *Dilwynites granulatus* Haris 1965, sample LMF025, EF Q9/4. 4) *Microcachryidites antarcticus* Cookson 1947 ex Couper 1953, sample LMF052, EF X36/1. 5) *Phyllocladidites mawsonii* (Cookson 1947) ex Couper 1953, sample LMF032, EF S42/2. 6) *Podocarpidites marwickii* Couper 1953, sample LMF025, EF Q47/1. 7) *Taxodiaceapollenites hiatus* (Potonie 1931) Kremp 1949, sample LMF032, EF S42/2. 8) *Podocarpidites otagoensis* Couper 1953, sample LMF020, EF H35/2. 9) *Podocarpidites* sp., sample LMF022, EF U41/3. 10) *Trisaccites* sp., sample LMF024, EF S13. 11) *Arecipites minutiscabratus* McIntyre 1968, sample LMF052, EF E51/3. 12) *Arecipites* sp. A Mildenhal &Pocknal 1989, sample LMF025, EF W14. 13) *Bombacacidites* sp., sample LMF025, EF H18/2-4. 14) cf. *Bacumorphomonocolpites* sp. Sole de Porta 1971, sample LMF025, EF P45/2. 15) *Diporites aspis* Pocknall & Mildenhall 1984, sample LMF051, EF F18/3. 16) *Horniella* sp. 2 Jaramilo & Dilcher 2001, sample LMF054, EF N39/3. 17, 18) *Ericipites* sp., sample LMF027, EF Y11/ 4; O42/4.

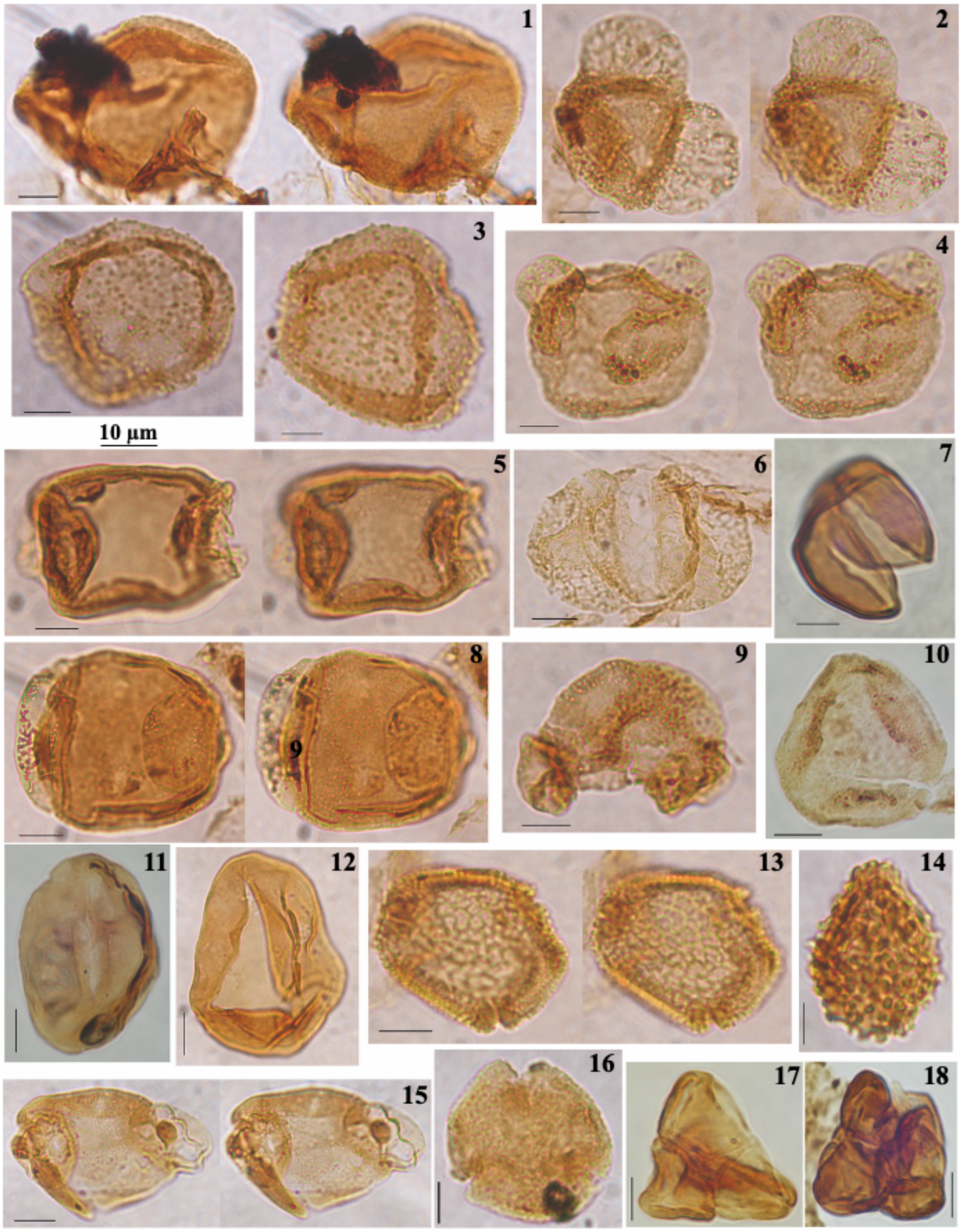

PLATE 4

1) *Favitricolporites* cf. *australis* Archangelsky 1973, sample LMF023, EF G7/2. 2) *Gothanipollis perplexus* Pocknal & Mildenhall 1984, sample LMF032, EF O10/3. 3) *Liliacidites* sp., sample LMF051, EF R14/1. 4) *Liliacidites* sp. 2 Archangelsky 1973, sample LMF 028, EF K21/1. 5) *Liliacidites variegatus* Couper 1953, sample LMF028, EF K40. 6) *Malvacipollis diversus* Harris 1965, sample LMF044, EF H31/1-2. 7) *Margocolporites* sp., sample LMF054, EF T24/1. 8) *Mauritiidites francisco* var. *minutus* Van der Hammen & Garcia 1966, sample LMF022, EF V9/1. 9) *Margocolporites* cf. *tenuireticulatus* Barreda 1997, sample LMF054, EF W33/4. 10) *Nothofagidites kaitangataensis* (Te punga) Romero 1973, sample LMF020, EF Q23/4. 11) *Nothofagidites dorotensis* Romero 1973, sample LMF024, EF W23/4. 12) *Nothofagidites acromegacanthus* Menéndez & Caccavari 1975, sample LMF021, EF R39/3. 13) *Nothofagidites* cf. *kaitangataensis* (Te punga) Romero 1973, sample LMF054 W12 4. 14) *Nothofagus fusca* group, sample LMF020, EF W32/4. 15) *Proteacidites* cf. *subscabratus* Couper 1960, sample LMF052, EF T51/2. 16) *Proteacidites* cf. *retiformis* Couper 1960, sample LMF054, EF J45/2. 17) *Proteacidites subscabratus* Couper 1960, sample LMF051, EF F12/1. 18) *Psilamonocolpites* sp., sample LMF028, EF P51/1-2.

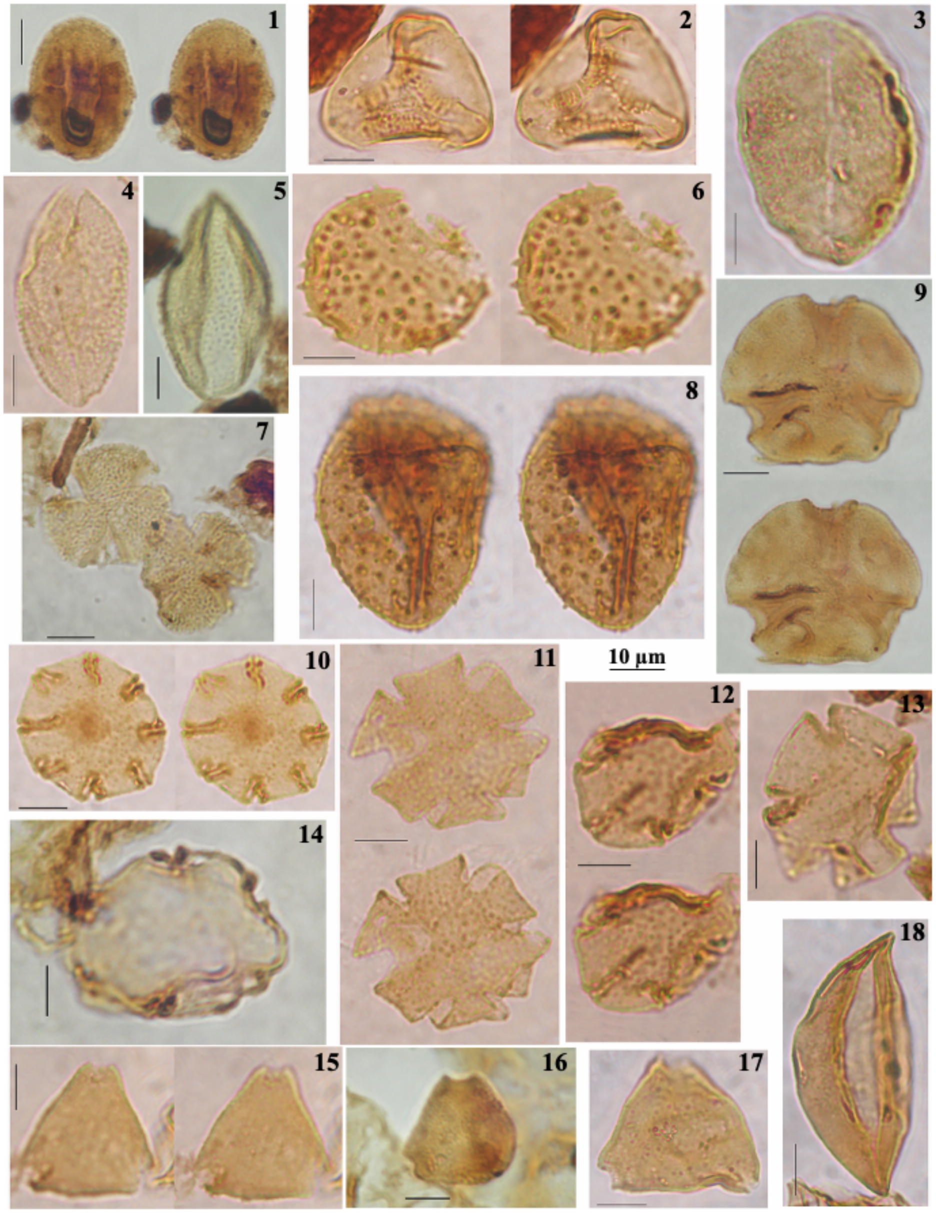

PLATE 5

1) *Psilatricolpites inargutus* (McIntyre 1968) Archangelsky 1973, sample LMF028, EF P38/4. 2) *Psilatricolporites* sp., sample LMF020, EF R46. 3) *Retistephanocolpites regularis* Hoeken-Klinkenberg 1966, sample LMF023, EF O39/3. 4) *Retitricolporites medius* González Guzmán 1967, sample LMF052, EF T12/2.

5) *Rhoipites* cf. *baculatus* Archangelsky 1973, sample LMFO52, EF W9/4. 6) *Rhoipites* sp., sample LMF054, EF S6/2. 7) *Tricolpites* cf. *reticulata* Cookson 1947, sample LMF024, EF F24/3-4. 8) *Rousea* cf. *microreticulata* Archangelsky & Zamaloa 1986, sample LMF027, EF F22/3. 9) *Tricolpites trioblatus* Mildenhall & Pocknall 1989, sample LMF026, EF F11/3-4. 10) *Striaticolporites gameroi* Archangelsky 1973, LMFO51, EF U32. 11) *Triorites minusculus* McIntyre 1965, sample LMF024, EF U42/4. 12, 13) Algae group, samples LMF020, LMF022, EF T28/2, EF N33/1. 14) Fungi, sample LMF024, EF L12/1-3. 15) *Tetraploa* sp., sample LMF023, EF H26.

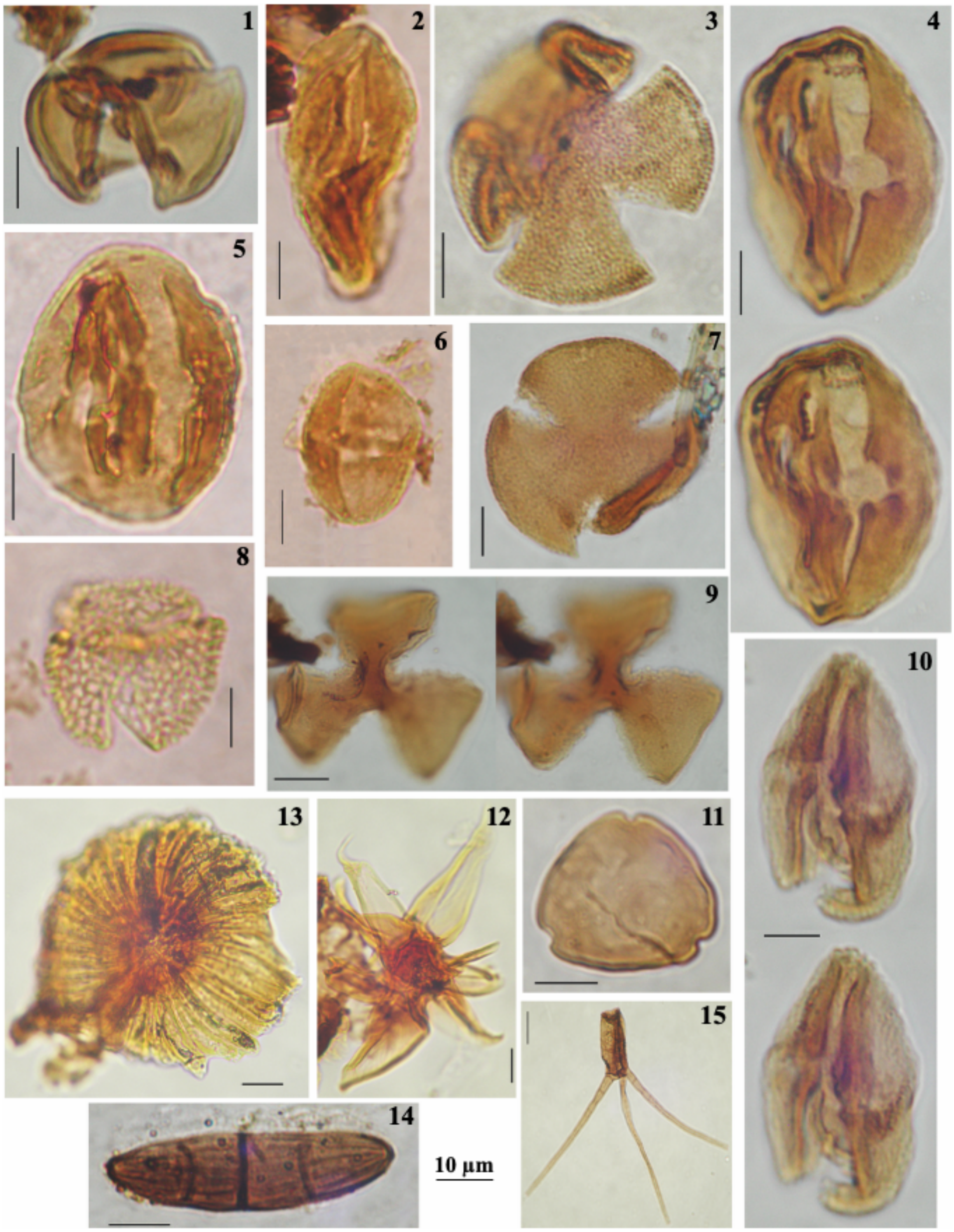

Figure S2. UPGMA Cluster analysis based on Sørensen Dissimilarity between this study and previously published work by Troncoso et al. 2000 and Macphail et al. 2013.

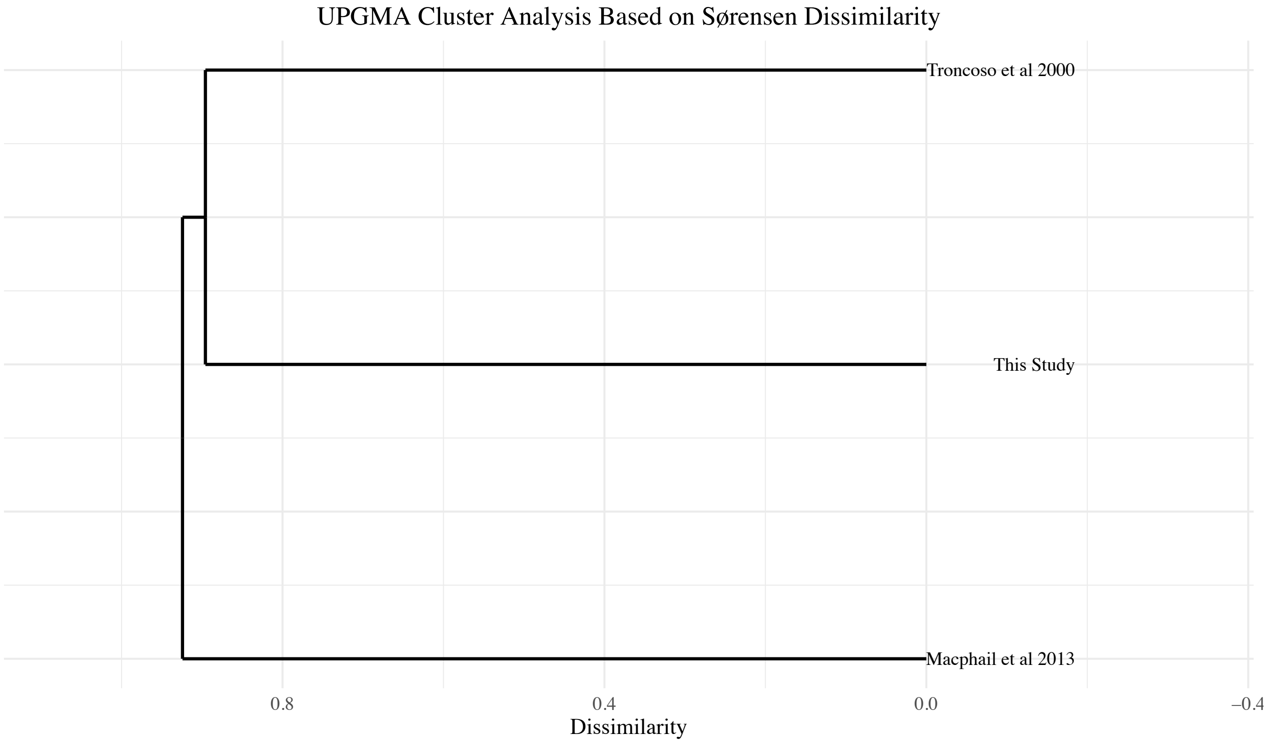

Figure S3: Köppen–Geiger reconstructions for the Southern Hemisphere. (A) Ensemble mean based on seven models under 3×CO_2. (B) Ensemble mean based on three models under 6×CO_2. During the Early Eocene, Antarctica was predominantly characterized by continental (D) climates, including Dsa and colder variants, whereas humid temperate Cf climates were spatially restricted.

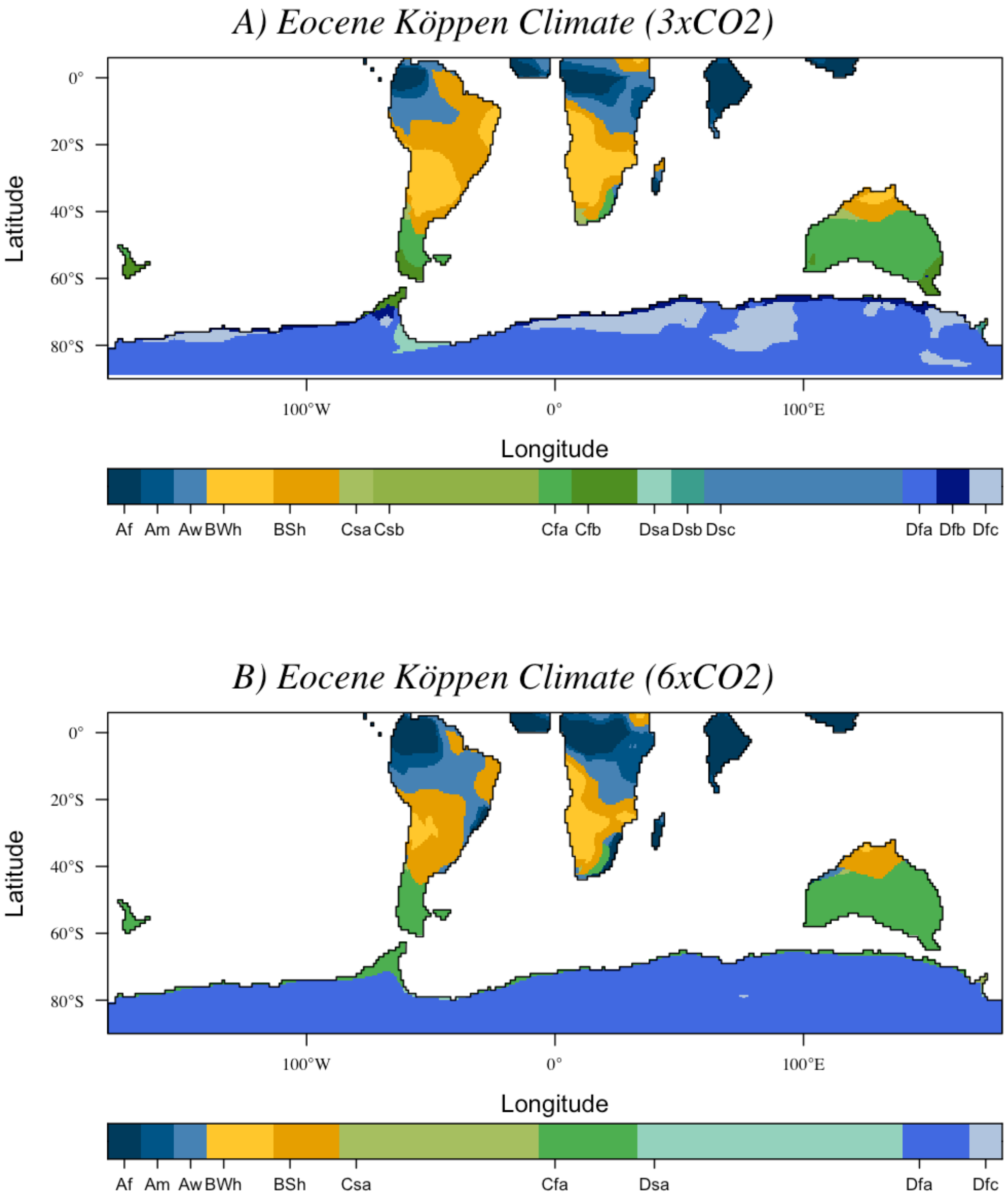

Figure S4: Modeled suitability for *Nothofagus* across Antarctica and the Australian region under (a) 3×CO_2 and (b) 6×CO_2 for Experiment 1 (modern-to-Eocene transfer), and under (c) 3×CO_2 and (d) 6×CO_2 for Experiment 2 (Eocene-to-Eocene calibration). The yellow dot marks the Wilkes Land offshore drill site (IODP Site U1356). Contours show mean sea-level pressure (hPa).

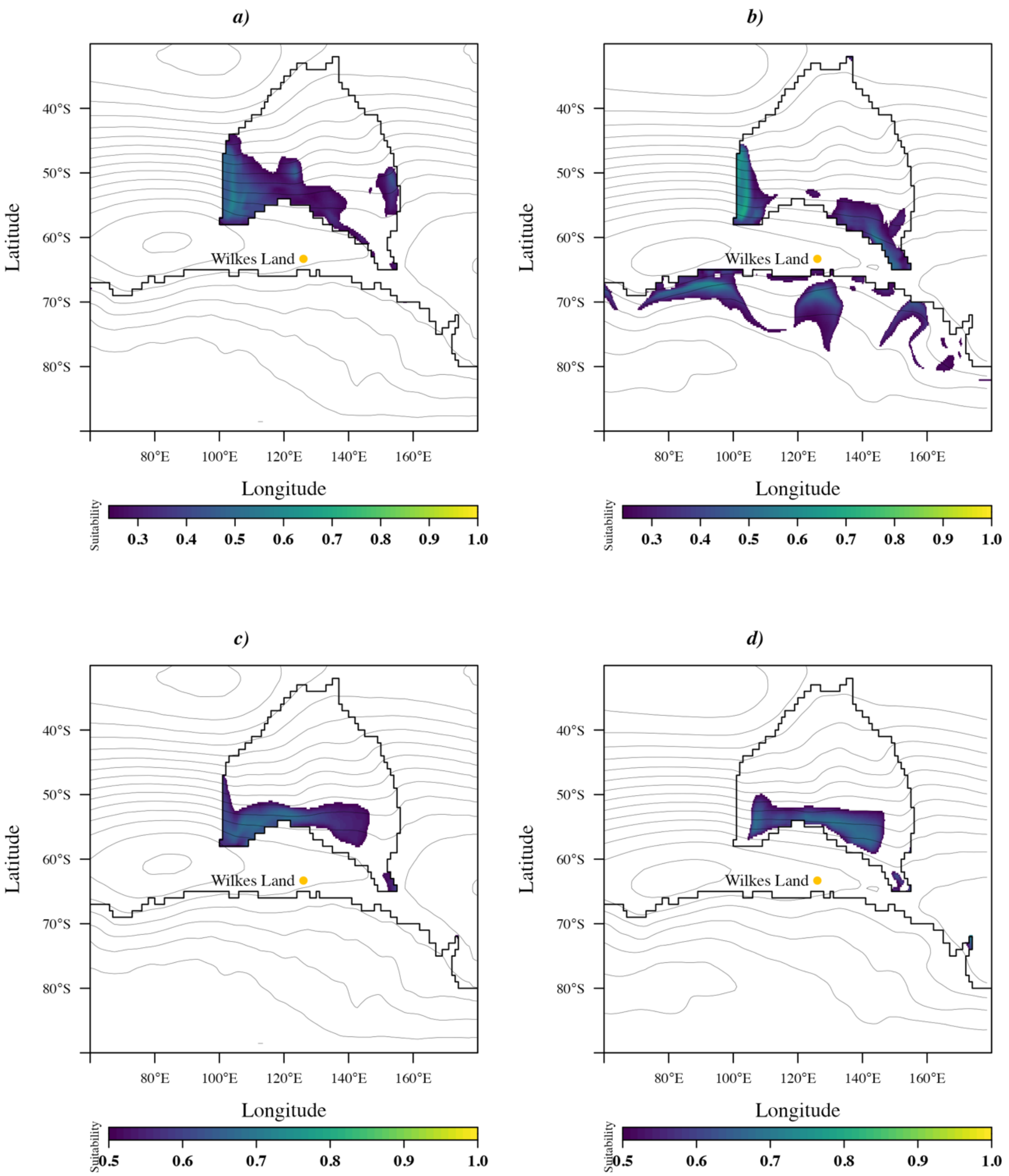

Figure S5: Rarefaction (continuous lines) and extrapolation (break lines) analysis comparing the Ligorio Marquez Formation with the Paleogene microflora of Rio Turbio, Laguna del Hunco, Agua Fresca, Chorrillo Chico, and the offshore core Wilkes Land. Shadow areas correspond to a 95% confidence interval.

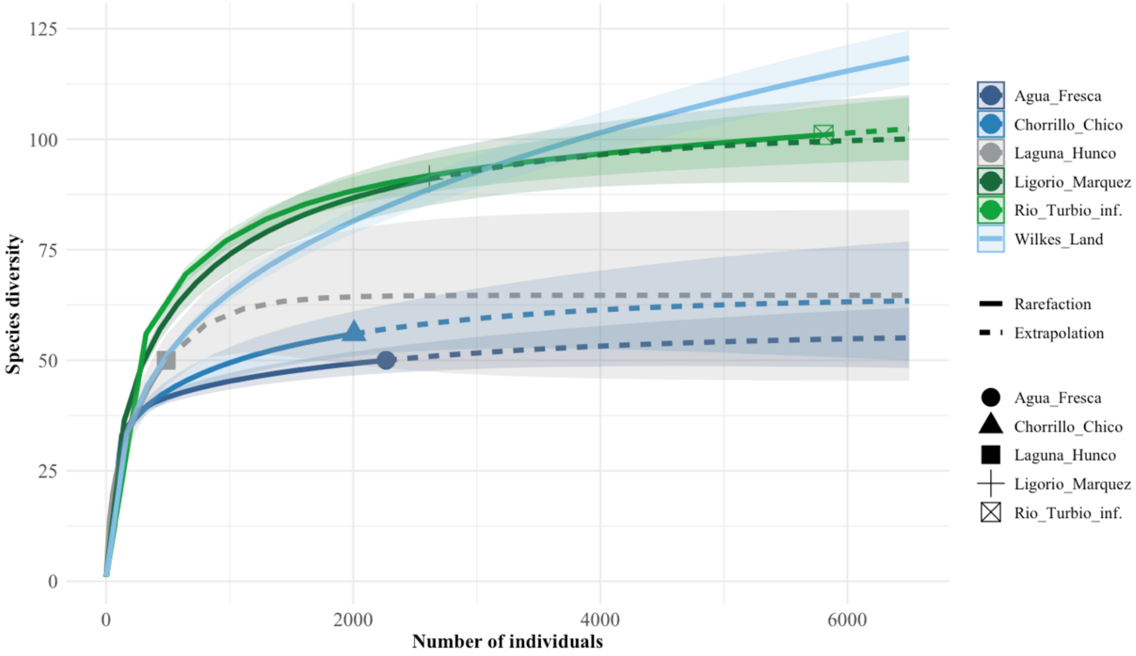

Figure S6: Ligorio Marquez Formation geological description and age discussion.

The LMF comprises three sedimentary units. The lower unit consists of dark grey siltstones interbedded with fine- to coarse-grained sand, occasionally containing granules. The central unit primarily comprises sand interspersed with fine pebble siltstones, which contain well-preserved plant remains. The sandy layers exhibit planar cross-stratification. The upper unit consists of carbonaceous siltstones with a few thin coal layers, suggesting a swamp environment. The abundance of fossil roots, particularly in sandstones with coal lines, indicates a predominantly continental depositional environment, likely a floodplain adjacent to river channels, represented by horizontally bedded sandstone layers (Yabe *et al.*, 2006). Also, Carvajal (2013) proposes a terrestrial origin for the formation exposed at "Mina Ligorio Márquez.". The LMF shows an angular unconformity with the underlying Upper Jurassic-Lower Cretaceous strata of the El Toqui Formation, a disconformity with Lower Cretaceous strata of the Divisadero Formation, and a disconformity with the overlying “Basaltos Inferiores Meseta Chile Chico (BIMCC) Formation(De la Cruz & Suárez, 2008; Encinas *et al.*, 2019).

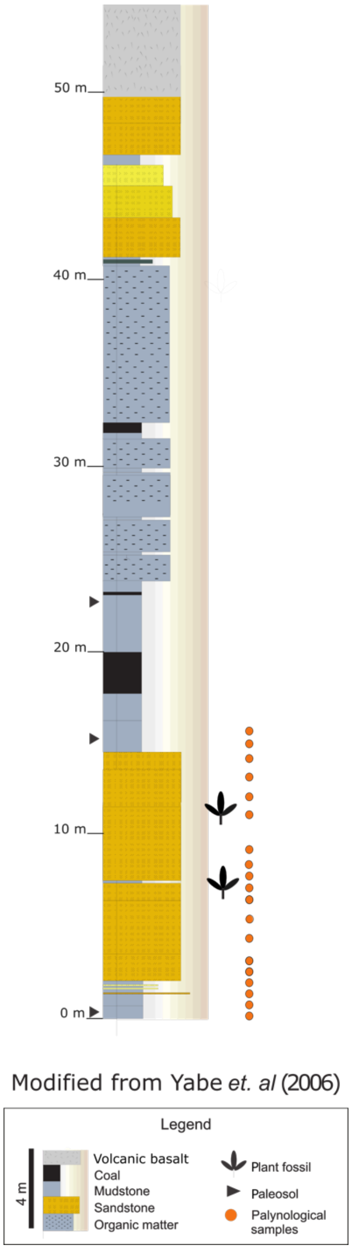

A total of 35 horizons were sampled along the 55-meter section (see column). Specifically, from base to top, sampling was conducted in floodplain and sandstone deposits (first 20 meters of the section), coal seams and siltstones (between 20 and 40 meters), and sandstones in the upper section (between 40 and 50 meters). The spacing between samples was irregular, depending on lithology and the sections covered. Most of the palynologically productive samples were recovered from the lowest stratigraphic level.

The high richness values at the bottom of the column may be due to a large contribution of extra local material from its depositional environment (a floodplain). However, the presence of fossil leaves in the first few meters of the column confirms the in-situ deposition of the material, and the presence of families such as Arecaceae, Bombacaceae, Ericaceae, and Fabaceae, pollinated by birds or insects, provides evidence of deposition restricted to an area of less than 100 meters (Bush & Rivera, 1998).

The age of the LMF is constrained by U-Pb zircon dating as well as K-Ar and Ar-Ar methods. According to Colwyn et al. (2019) three U-Pb zircon ages obtained from stratigraphically

ascending levels in LMF are 57.3 ± 2.7 Ma, 53.7 ± 2.9 Ma, and 50.5 ± 2.5 Ma. The first two ages suggest a maximum depositional age ranging from the late Paleocene (Thanetian) to the early Eocene (Ypresian), while the youngest age constrains the upper part of the Formation to the early Eocene. This latter age is statistically indistinguishable from the radiometric date of 47.6 ± 0.78 Ma (K-Ar; (Yabe *et al.*, 2006)) and 53.4 ± 0.2 Ma ($^{40}Ar/^{39}Ar$;(Encinas *et al.*, 2019)) obtained from the overlying basalt of the BIMCC. Accordingly, the age of LMF will be between Late Paleocene (Thanetian) and Early Eocene (Ypresian).

The age of the flora has been approached by different authors. For example, Suárez et al. (2000) and Troncoso *et al. (2002)* reported 19 leaf taxa, mainly associated with Lauraceae, and 12 spore and pollen taxa, highlighting the absence of the iconic Gondwana taxa, *Nothofagus*. These authors compared their findings with the Chilean fossil flora of Lota-Coronel (Engelhardt, 1891) and suggested a similar age. Lota-Coronal flora is deposited in the coal-bearing strata of the Curanilahue Formation in Central Chile, with an age of Upper Paleocene-Early Eocene (but see (Carpenter & Mcloughlin, 2025). U-Pb zircons on the base of Curanilahue Formation give a maximum age of 57 Ma (Zambrano *et al.*, 2014) and a minimum age of 52.3 – 50.8 Ma for the presence of the planktonic foraminifer *Morozovella aragonensis* in the overlain Boca-Lebu Formation (Le Roux & Elgueta, 1997; Berggren & Pearson, 2005). Macphail *et al.* (2013) reported *Dilwynites* (Wollemia-type pollen) along with 22 other pollen taxa from Argentinian outcrops of the LMF. The authors suggest that their samples are most likely early Eocene in age and no younger than middle Eocene, based on the absence of *Nothofagus* in the samples. Our previous work on the LMF has yielded leaf and pollen fossils associated with *Nothofagus* in the flora (Hinojosa *et al.*, 2016). The occurrence of *Malvacipollis diversus* and *Retitricolporites medius* in the palynoflora suggests an Early Eocene age for the LMF flora(Carvajal, 2013; Hinojosa *et al.*, 2016; Quattrocchio *et al.*, 2024), in agreement with the radiometric ages reported for the Formation.

Table S1: Ligorio Marquez palynoflora. Include the pollen identified in Troncoso et al 2000 and Macphail et al 2013.

**Ligorio Marquez Spores and Pollen**	**This Study**	**Troncoso et al 2000**	**Macphail et al 2013**
Baculatisporites comaumensis (Cookson 1953) Potonie 1956	1	0	0
Baculatisporites turbioensis Archangelsky 1972	1	0	0
Baculatisporites sp.	1	0	0
Biretisporites sp.II Archangelsky 1972	1	0	0
Biretisporites cf. crassilabratus Archangelsky 1972	1	0	0
Biretisporites crassilabratus Archangelsky 1972	1	0	0
Biretisporites sp.	1	0	0
Cingutriletes australis (Cookson) Archangelsky 1972	1	0	0
Clavifera triplex	0	1	0
Concavisporites sp.	1	0	0
Corrugatisporites cf. argentinus Archangelsky 1972	1	0	0
Cyathidites minor Couper 1953	0	1	0
Cyathidites sp.	0	0	1
Cyatheacidites cf. annulatus Cookson 1947	1	0	0
Deltoidospora minor (Couper) Pocock 1970	1	0	0
Deltoidospora sp.	1	0	0
Dictyophyllidites pectinataeformis (Bolkhovitina) Dettman 1963	1	0	0
Dictyophyllidites cf. crenatus Dettman 1963	1	0	0
Dictyophylliidites sp.	1	0	0
Echinatisporis sp.	1	0	0
Ischyosporites cf. crateris Balme 1957	1	0	0
Ischyosporites sp.	1	0	0
Ischyosporites areapunctata (Stuchlik) Barreda	0	0	1
Kluklisporites sp.	1	0	0
Phyllocladidites mawsonii Cookson ex Couper	0	0	1
Laevigatosporites ovatus Wilson & Webster 1946	1	1	0
Leiotriletes regularis (Pflug) Krutzsch 1959	1	1	0
Leptolepidites cf. verrucatus Couper 1953	1	0	0
Peromonolites sp.	1	0	0
Peromonolites vellosus Partridge 1973	1	0	0
Polypodiisporites spp.	1	0	0
Pseudoschizaea circula (Wolff) Christopher 1976	1	0	0
Reboulisporites fuegiensis Zamaloa & E.J. Romero	0	0	1
Retitriletes sp.	1	0	0
Trilites cf. parvallatus Krutzsch 1959	1	0	0
Verrucatitriletes sp.	1	0	0

Verrucosisporites sp.	0	1	0
Araucariacites australis Cookson 1947	1	0	0
Araucariacites sp.	1	0	0
Dacrycarpites australiensis Cookson & Pike 1953	1	0	1
Dacrydiumites florinii Cookson & K.M. Pike var.	0	0	1
Dilwynites granulatus Harris 1965	1	0	0
Dilwynites tuberculatus Harris 1965	1	0	1
Inaperturopollenites spp.	1	0	0
Microcachryidites antarcticus Cookson 1947 ex Couper 1953	1	0	1
Phyllocladidites mawsonii (Cookson 1947) ex Couper 1953	1	0	0
Podocarpidites marwickii Couper 1953	1	1	1
Podocarpidites otagoensis Couper 1953	1	1	0
Podocarpidites sp.	1	0	0
Podosporites microsaccatus (Couper) M.E. Dettmann	0	0	1
Taxodiaceapollenites hiatus (Potonie 1931) Kremp 1949	1	0	0
Trisaccites sp.	1	0	0
Ailanthipites sp.	0	0	1
Arecipites minutiscabratus McIntyre 1968	1	0	0
Arecipites spp. A Mildenhall & Pocknall 1989	1	0	0
Arecipites spp.	1	0	0
Bombacacidites sp.	1	0	1
cf. Bacumorphomonocolpites spp. Sole de Porta 1971	1	0	0
cf. Horniella spp. 2 Jaramillo & Dilcher 2001	1	0	0
Corsinipollenites sp.	1	0	0
Diporites aspis Pocknall & Mildenhall 1984	1	0	0
Ericipites microverrucatus	0	0	1
Ericipites sp. 1	1	0	0
Ericipites sp. 2	1	0	0
Favitricolporites cf. australis Archangelsky 1973	1	0	0
Gothanipollis perplexus Pocknall & Mildenhall 1984	1	0	0
Haloragacidites harrisii (Couper 1953) Harris 1971	0	1	0
Liliacidites sp. 1	1	0	0
Liliacidites sp. 2 Archangelsky 1973	1	0	0
Liliacidites variegatus Couper 1953	1	0	0
Liliacidites cf. L. regularis Archangelsky (Liliaceae)	0	0	1
Luminidites sp.	0	0	1
Malvacipollis diversus Harris 1965	1	0	0
Margocolporites cf. tenuireticulatus Barreda 1997	1	0	0
Margocolporites sp.	1	0	0

Mauritiidites franciscoi var. minutus Van der Hammen & Garcia 1966	1	0	0
Mutisiapollis sp.	0	0	1
Nothofagidites fusca group	1	0	0
Nothofagidites acromegacanthus Menéndez y Caccavari 1975	1	0	0
Nothofagidites dorotensis Romero 1973	1	0	0
Nothofagidites kaikangataensis (Te punga)Romero 1973	1	0	0
Nothofagidites kaikangataensis (Te punga)Romero 1973	1	0	0
Polyporina cf. romeroi	1	0	0
Proteacidites cf. retiformis Couper 1960	1	0	0
Proteacidites cf. subscabratus Couper 1960	1	0	0
Proteacidites subscabratus Couper 1960	1	0	0
Proteacidites parvus Cookson	0	1	0
Proteacidites sp.	0	0	1
Proxapertites sp.	1	0	0
Psilamonocolpites sp.	1	0	0
Psilatricolporites sp.	1	0	0
Psilatricolpites inargutus (McIntyre 1968) Archangelsky 1973	1	0	0
Retistephanocolpites regularis Hoeken-Klinkenberg 1966	1	0	0
Retistephanocolpites sp.	1	0	0
Retitricolporites medius González Guzmán 1967	1	1	0
Retitricolporites sp.	1	0	0
Rhoipites cf. baculatus Archangelsky 1973	1	0	0
Rhoipites sp.	1	0	0
Rousea cf. microreticulata Archangelsky & Zamaloa 1986	0	0	1
Schizocolpus sp.	0	0	1
Spinitricolpites sp	0	0	1
Stephanocolpites sp.	1	0	0
Striatricolporites gamerroi Archangelsky 1973	1	0	0
Tricolpites cf. reticulata Cookson 1947	1	0	0
Tricolpites trioblatus Mildenhall & Pocknall 1989	0	1	0
Tricolpites communis Archangelsky 1973	0	0	1
Tricolporites sp.	1	0	0
Triorites minusculus McIntyre 1965	0	0	1
Triprojectacites group cf. Integricorpus sp.	0	1	0

Table S2: Microflora of the Ligorio Marquez Formation and their botanical affinities, including the frequency of each morphotaxon used for rarefaction/extrapolation analysis.

No.	Ligorio Marquez Spores and Pollen	Botanical affinity	Frequency
1	Baculatisporites comaumensis (Cookson 1953) Potonie 1956	Hymenophyllaceae (Hymenophyllum sp.)	12
2	Baculatisporites turbioensis Archangelsky 1972	Osmundaceae	2
3	Baculatisporites sp.	Unknown trilete spore	7
4	Biretisporites sp.II Archangelsky 1972	?Hymenophylleaeceae	23
5	Biretisporites cf. crassilabratus Archangelsky 1972	Osmundaceae	4
6	Biretisporites crassilabratus Archangelsky 1972	Hymenophylleaeceae?	22
7	Biretisporites sp.	Unknown trilete spore	86
8	Cingutriletes australis (Cookson) Archangelsky 1972	Sphagnales (Sphagnum sp.)	2
9	Concavisporites sp.	Filicopsida?	5
10	Corrugatisporites cf. argentinus Archangelsky 1972	Schizaeaceae	1
11	Cyatheacidites cf. annulatus Cookson 1947	Lophosoriaceae (Lophosoria sp.)	6
12	Deltoidospora minor (Couper) Pocock 1970	Polypodiaceae (Acrostichum sp.)	151
13	Deltoidospora sp.	Polypodiaceae	48
14	Dictyophyllidites pectinataeformis (Bolkhovitina) Dettman 1963	Matoniaceae (Matonia pectinata)	3
15	Dictyophyllidites cf. crenatus Dettman 1963	Matoniaceae	1
16	Dictyophylliidites sp.	Matoniaceae	5
17	Echinatisporis sp.	Selaginellaceae (Selaginella sp.)	1
18	Ischyosporites cf. crateris Balme 1957	Dicksoniaceae (Dicksonia sp.)	8
19	Ischyosporites sp.	Dicksoniaceae	20
20	Kluklisporites sp.	Schizaeaceae	40
21	Laevigatosporites ovatus Wilson & Webster 1946	Blechnaceae	33
22	Leiotriletes regularis (Pflug) Krutzsch 1959	Schizaeaceae / Matoniaceae?	10
23	Leptolepidites cf. verrucatus Couper 1953	Lycopsida (Leptolepia sp.)	4
24	Peromonolites sp.	Blechnaceae/ Dryopteridaceae	5
25	Peromonolites vellosus Partridge 1973	Blechnaceae	2
26	Polypodiisporites spp.	Polypodiaceae	33
27	Pseudoschizaea circula (Wolff) Christopher, 1976	Zygnemataceae	19
28	Retitriletes sp.	Lycopodiaceae	7
29	Trilites cf. parvallatus Krutzsch 1959	Dicksoniaceae (Dicksonia)	1
30	Verrucatitriletes sp.	Unknown trilete spore	17
31	Araucariacites australis Cookson, 1947	Araucariaceae (Araucaria)	35
32	Araucariacites sp.	Araucariaceae	1
33	Dacrycarpites australiensis Cookson & Pike 1953	Podocarpaceae	25
34	Dilwynites granulatus Harris 1965	Araucariaceae	392
35	Dilwynites tuberculatus Harris 1965	Araucariaceae	7

36	Inaperturopollenites spp.	Unknown Gimnosperm	41
37	Microcachryidites antarcticus Cookson 1947 ex Couper 1953	Podocarpaceae (Microcachrys)	56
38	Phyllocladidites mawsonii (Cookson 1947) ex Couper 1953	Podocarpaceae (Dacrydium franklinii)	5
39	Podocarpidites marwickii Couper 1953	Podocarpaceae	211
40	Podocarpidites otagoensis Couper 1953	Podocarpaceae	19
41	Podocarpidites sp.	Podocarpaceae	727
42	Taxodiaceaepollenites hiatus (Potonie 1931) Kremp 1949	Taxodiaceae	13
43	Trisaccites sp.	Podocarpaceae	8
44	Arecipites minutiscabratus McIntyre 1968	Arecaceae	5
45	Arecipites spp. A Mildenhall & Pocknall 1989	Arecaceae	5
46	Arecipites spp.	Arecaceae	4
47	Bombacacidites sp.	Bombacaceae	3
48	cf. Bacumorphomonocolpites spp. Sole de Porta 1971	Unknown angiosperm	2
49	cf. Horniella spp. 2 Jaramillo & Dilcher 2001	Unknown angiosperm	1
50	Corsinipollenites sp.	Onagraceae	1
51	Diporites aspis Pocknall & Mildenhall 1984	Onagraceae (Fuchsia sp.)	1
52	Ericipites sp. 1	Ericaceae/Empetraceae	37
53	Ericipites sp. 2	Ericaceae/Empetraceae	7
54	Favitricolporites cf. australis Archangelsky 1973	Unknown angiosperm	2
55	Gothanipollis perplexus Pocknall & Mildenhall 1984	Loranthaceae (Elytranthe blume)	3
56	Liliacidites sp. 1	Liliaceae	1
57	Liliacidites sp. 2 Archangelsky 1973	Liliaceae	10
58	Liliacidites variegatus Couper 1953	Liliaceae	5
59	Malvacipollis diversus Harris 1965	Malvaceae	1
60	Margocolporites cf. tenuireticulatus Barreda 1997	Unknown angiosperm	4
61	Margocolporites sp.	Fabaceae	2
62	Mauritiidites franciscoi var. minutus Van der Hammen & Garcia 1966	Arecaceae	4
63	Nothofagidites fusca group	Nothofagaceae	3
64	Nothofagidites acromegacanthus Menéndez y Caccavari 1975	Nothofagaceae	9
65	Nothofagidites dorotensis Romero 1973	Nothofagaceae	46
66	Nothofagidites kaikangataensis (Te punga)Romero 1973	Nothofagaceae	23
67	Nothofagidites kaikangataensis (Te punga)Romero 1973	Nothofagaceae	17
68	Polyporina cf. romeroi	Unknown angiosperm	2
69	Proteacidites cf. retiformis Couper 1960	Proteaceae	2
70	Proteacidites cf. subscabratus Couper 1960	Proteaceae	1
71	Proteacidites subscabratus Couper 1960	Proteaceae	2

72	Psilamonocolpites sp.	Unknown angiosperm	35
73	Psilatricolporites sp.	Unknown angiosperm	4
74	Psilatricolpites inargutus (McIntyre 1968) Archangelsky 1973	Violaceae?	1
75	Retistephanocolpites regularis Hoeken-Klinkenberg 1966	Bombacaceae (Catostemma sp.)	1
76	Retistephanocolpites sp.	Unknown angiosperm	3
77	Retitricolporites medius González Guzmán 1967	Unknown angiosperm	2
78	Retitricolporites sp.	Unknown angiosperm	5
79	Rhoipites cf. baculatus Archangelsky 1973	Rutaceae /Araliaceae?/Mimosaceae	2
80	Rhoipites sp.	Unknown angiosperm	8
81	Rousea cf. microreticulata Archangelsky & Zamaloa 1986	Unknown angiosperm	3
82	Striatricolporites gamerroi Archangelsky 1973	Solanaceae/Rosaceae(Fragaria sp.)	1
83	Tricolpites cf. reticulata Cookson 1947	Gunneraceae (Gunnera sp.).	20
84	Tricolpites trioblatus Mildenhall & Pocknall 1989	Scrophulariaceae/Convolvulaceae	1
85	Triorites minusculus McIntyre 1965	Unknown angiosperm	25
85	Algae spore type 1	Unknown algae	3
86	Algae spore type 2	Unknown algae	9
87	*Tetraploa* sp.	Unknown fungi	7
88	Fungi spore	Unknown fungi	19
89	Indeterminate Pollen	Unknown	106
90	Indeterminate Spore	Unknown	40

Table S3: Assemblage-level sampling effort and richness (N, S_obs, and S(m0) with 95% bootstrap CIs) between Ligorio Marquez Formation and Paleogene Patagonian palynofloras. N number of individuals, Sobs Total palynomorph species, m_0 rarefied richness, CI95 bootstrap 95% confidence interval.

**Assemblage**	**N**	**Sobs**	**m_0**	**S_m_0**	**CI95_low**	**CI95_high**
Chorrillo_Chico	2007	56	2007	51.87	48.98	55.00
Agua_Fresca	2266	50	2007	46.88	44.53	49.11
Ligorio_Marquez	2616	91	2007	80.48	75.81	84.68
Rio_Turbio_low.	5807	101	2007	85.6	83.01	87.86
Rio_Turbio_upp.	7502	107	2007	84.23	80.79	86.81
Laguna del Hunco	486	50	-	-	-	-

Reference Supporting Information

Berggren WA, Pearson PN. 2005. A revised tropical to subtropical paleogene planktonic foraminiferal zonation. *Journal of Foraminiferal Research* **35**(4): 279-298.

Bush MB, Rivera R. 1998. Pollen dispersal and representation in a neotropical rain forest. *Global Ecology and Biogeography* **7**(5): 379-392.

Carpenter RJ, Mcloughlin S. 2025. A new leaf species of Proteaceae and other Gondwanan elements from the early Paleogene Lota-Coronel flora of south-central Chile. *Australian Systematic Botany* **38**(1).

Carvajal F. 2013. *Paleoclima y Diversidad en la Palinoflora de la Formación Ligorio Marquez.* Master Thesis Master, Universidad de Chile Santiago.

Colwyn DA, Brandon MT, Hren MT, Hourigan J, Pacini A, Cosgrove MG, Midzik M, Garreaud RD, Metzger C. 2019. Growth and Steady State of the Patagonian Andes. *American Journal of Science* **319**(6): 431-472.

De la Cruz R, Suárez M 2008. Geología del Área de Chile Chico-Río de Las Nieves, Región de Aisén del General Carlos Ibáñez del Campo.In Chile CGd. *Serie Geología Básica, vol. 112,*. Santiago, Chile: Servicio Nacional de Geología y Minería.

Encinas A, Folguera A, Riffo R, Molina P, Paz LF, Litvak VD, Colwyn DA, Valencia VA, Carrasco M. 2019. Cenozoic basin evolution of the Central Patagonian Andes: Evidence from geochronology, stratigraphy, and geochemistry. *Geoscience Frontiers* **10**(3): 1139-1165.

Engelhardt H. 1891. Über Tertiärpflanzen von Chile. *Abhandlungen der Senckenbergischen Naturforschenden Gessellschaft* **16**: 629-692.

Hinojosa LF, Gaxiola A, Perez MF, Carvajal F, Campano MF, Quattrocchio M, Nishida H, Uemura K, Yabe A, Bustamante R, et al. 2016. Non-congruent fossil and phylogenetic evidence on the evolution of climatic niche in the Gondwana genus Nothofagus. *Journal of Biogeography* **43**(3): 555-567.

Le Roux JP, Elgueta S. 1997. Paralic parasequences associated with Eocene sea-level oscillations in an active margin setting: Trihueco formation of the Arauco basin, Chile. *Sedimentary Geology* **110**(3-4): 257-276.

Macphail M, Carpenter RJ, Iglesias A, Wilf P. 2013. First Evidence for Wollemi Pine-type Pollen (Dilwynites: Araucariaceae) in South America. *Plos One* **8**(7).

Quattrocchio M, Aguero S, Iglesias A, Raigemborn MS. 2024. Palynostratigraphy of the early Paleogene at the Laguna Manantiales locality, southern Golfo San Jorge Basin, Argentina. *Publicación Electrónica de la Asociación Paleontológica Argentina* **24**(1): 108-128.

Suárez M, de la Cruz R, Troncoso A. 2000. Tropical/Subtropical upper Paleocene - lower Eocene fluvial deposits in eastern central Patagonia, Chile (46°45'S). *Journal of South American Earth Sciences* **13**: 527-536.

Troncoso A, Suárez M, de la Cruz R, Palma-Heldt S. 2002. Paleoflora de la Formación Ligorio Márquez (XI Región, Chile) en su localidad tipo: sistemática, edad e implicancias paleoclimáticas. *Revista Geológica de Chile* **29**(Nº 1): 113-135.

Yabe A, Uemura K, Nishida H 2006. Geological notes on plant fossil localities of the Ligorio Márquez Formation, central Patagonia, Chile. In: Nishida H ed. *Post-Creataceus floristic changes in southern Patagonia, Chile*. tokyo, Japan: Chuo University, 29-35.

Zambrano P, Encinas A, Buatois L, Arenillas I, Stinnesbeck W, Nielsen SN 2014. Sedimentology, age and provenance of Paleogene delta systems from Central Chile. *XIV Reunión Argentina de Sidementología*. Puerto Madryn, Argentina. 299-300.
